# High energy resolution fluorescence detected X-ray absorption spectroscopy (HERFD-XAS) for studies of metals and metalloids in biology: current innovations and future perspectives

**DOI:** 10.1093/mtomcs/mfaf038

**Published:** 2025-11-14

**Authors:** Ani T Baker, Graham N George, Hugh H Harris

**Affiliations:** Discipline of Chemistry, The University of Adelaide, North Terrace, Adelaide, SA 5005, Australia; Department of Geological Sciences, Department of Chemistry and Toxicology Center, University of Saskatchewan, 114 Science Place, Saskatoon, SK S7N 5E2, Canada; Discipline of Chemistry, The University of Adelaide, North Terrace, Adelaide, SA 5005, Australia

## Abstract

X-ray absorption spectroscopy (XAS) is a technique which is frequently used in metallomics research, providing a valuable tool for the elucidation of element-specific electronic and geometric structural information. Recent decades have seen the development of related synchrotron-based X-ray techniques with enhanced analytical capabilities, including X-ray emission spectroscopy (XES), resonant inelastic X-ray scattering (RIXS), and high energy resolution fluorescence detected X-ray absorption spectroscopy (HERFD-XAS). With appropriate experimental configuration, HERFD-XAS can generate spectra with significantly improved spectroscopic resolution and background rejection compared to conventional XAS, providing a substantial advantage in the analysis of dilute analytes in biological samples. These improvements arise from the capability to interrogate selected fluorescence lines with the use of multiple crystal analyzers, minimizing the effects of core-hole lifetime broadening. Herein, we review a range of existing and emerging applications of HERFD-XAS for the study of metals and metalloids in biology and medicine. Direct comparisons of conventional XAS and HERFD-XAS spectra highlight the substantial improvements in resolution, and greater potential for the interpretation of metal speciation in complex and dilute biological samples. We also discuss current challenges with the design of HERFD-XAS experiments.

## Graphical Abstract

**Graphical Abstract:**
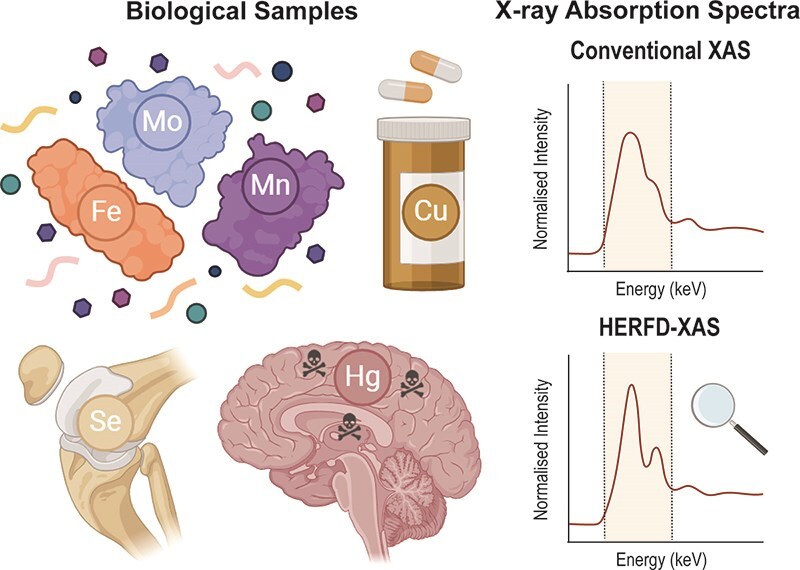

## Introduction: the role of the metallome

The biosphere availability of metal ions has fluctuated significantly across geological time [1], driving the evolution of sequestration and efflux systems for metal ion regulation in organisms. The distribution of metals and metalloids in an organism is often termed the ‘metallome’, collectively referring to the aqueous and ligand-bound metal content in cells, with the ensemble of metals complexed with biomolecules, particularly proteins, collectively establishing the ‘metalloproteome’ [2]. The study of metallomics is a dynamic field of research, encompassing the analysis of metal ions at the scale of organs, tissues, cells and proteins, and their interaction with other components in these systems [3].

Metals essential for human life and health are often divided into two categories; (i) the four group one metals, sodium (Na), potassium (K), magnesium (Mg), and calcium (Ca), which are essential for the maintenance of electrochemical gradients, cell signaling and utilized as enzyme cofactors, and (ii) the six *d*-block transition metals, manganese (Mn), iron (Fe), cobalt (Co), copper (Cu), zinc (Zn), and molybdenum (Mo), which perform catalytic actions, facilitate electron transfer reactions, and exist within proteins which counteract oxidative stress, among many other roles [4]. The ‘bulk’ essential elements including carbon (C), hydrogen (H), nitrogen (N), oxygen (O), phosphorus (P), and sulfur (S), provide structural complexity and permit the generation of large biomolecules constructed from stable covalent bonds, however, these elements are incapable of supporting life alone [4,5]. Metal ions provide organisms with access to chemistry which is unachievable utilizing only the ‘bulk’ atoms and introduce molecular complexity through the incorporation of coordinate bonds [6].

The importance of this chemical complexity to the maintenance of life is reflected in the variety of metals present across all classes of biomolecules, particularly in metalloproteins. However, the distribution of metals at various levels (i.e. within cells, tissues, and organs) must be tightly regulated to uphold a sensitive balance between essentiality and toxicity. In many cases, the consequences of perturbations in the homeostasis of these essential trace metals can be severe, resulting in diseases such as pernicious anemia, growth retardation, and heart disease, owing to deficiencies in Fe, Zn, and Cu, respectively [7]. Excess or deficit of these metals (and other essential elements) are also associated with many additional neurodegenerative and cardiovascular diseases, diabetes, and cancer [8].

Amongst the less well-recognized essential roles of metals, Co fulfills a range of critical roles in many organisms, typically incorporated as cobamides or corrinoid heterocycles (e.g. vitamin B12 and coenzyme B12) [9,10], but also exists without association with the B12 cofactor, in enzymes such as alternate carbonic anhydrases [11,12], nitrile hydratase [13,14], and methionine aminopeptidase [15]. Beyond this, several studies have reported beneficial biological effects of first-row non-essential transition metals vanadium (V) and nickel (Ni), demonstrating their associations with various proteins and related cellular redox processes [16,17]. V is recognized as an essential component of the cofactor of several nitrogenases including those found in the prokaryotes *Azotobacter vinelandii, Methanosarcina acetivorans*, and *Rhodopseudomonas palustri*, all of which possess genes for Mo-, Fe-, and V-dependent nitrogenases [4,18–20]. Similarly, the role of a Ni(II) cofactor in the active site of hydrogenases, carbon monoxide dehydrogenases and ureases in a range of organisms, including several human pathogens, is well established [21–23].

Generally considered a toxic heavy metal, cadmium (Cd) has been shown to exhibit biological utility through its incorporation into carbonic anhydrase in marine diatoms [4,24,25], and although more obscure relative to first-row metal-containing enzymes, there have been many tungsten (W) enzymes identified in prokaryotes, including hyperthermophilic archaea, methanogens, and acetogens, where these W enzymes typically serves to replace Mo-containing homologs [26,27]. These Mo- or W-containing enzymes include aldehyde oxidoreductases [26,28], formate dehydrogenases, and formylmethanofuran dehydrogenases, with the latter enzyme performing archaeal methanogenesis—the most ancient method of carbon dioxide fixation [29].

Although there is no definitively known essential role for titanium (Ti) in biology, some marine invertebrates (tunicates) are reported to sequester high concentrations of Ti, either selectively or in conjunction with other metal ions (commonly V) [30]. Similarly, marine diatoms (e.g. sea plankton) have been observed to sequester Ti from growth media [30,31], and recent studies have demonstrated the bioaccumulation of Ti within the protective outer cell wall (frustules) of freshwater diatoms [32]. Recent studies have also reported the emerging and potential roles of rare earth metals in biological processes [33], with evidence to support the functional role of lanthanides as a cofactor with pyrroloquinoline quinone in bacterial methanol and ethanol dehydrogenases [34,35].

Collectively, these findings emphasize the significance of metal ions in biology and their vast range of roles in organisms, many of which remain unrecognized, or their mechanisms of action poorly understood. Furthermore, several metals typically considered non-essential to life, including ruthenium (Ru), rhodium (Rh), platinum (Pt), and gold (Au), are utilized for pharmacological applications in the design of therapeutic agents, such as cisplatin (Pt) and auranofin (Au) [36,37], which must be carefully characterized in biological environments before clinical use.

With the current and emerging capabilities of high-resolution X-ray spectroscopy methods, we may begin to uncover new information about previously considered non-essential metal ions and their utility in biology and medicine. However, this research can be inherently challenging as the treatment of samples must be carefully considered to avoid destruction of the native state of the metallome. Challenges also arise due to the dilute nature of metal ions in many biological samples, and the matrix of other components surrounding the molecule of interest. Overcoming these obstacles requires direct and sensitive experimental methods.

## X-ray absorption spectroscopy methods

In this article, we describe the current state-of-play in the analytical capabilities of high energy resolution fluorescence detected X-ray absorption spectroscopy (HERFD-XAS), an experimental method which utilizes the sensitivity of a synchrotron light source to probe metal ions in intact biological systems at biologically relevant concentrations. We focus on the applications of this emerging method in complex biological systems, selecting examples which highlight the capabilities of HERFD-XAS to yield chemical insight for a range of metals at high dilution, particularly where this demonstrates an improvement in spectroscopic resolution when compared to conventional X-ray absorption spectroscopy (XAS). Preceding these topics, we will first cover the underlying concepts of synchrotron-based X-ray techniques, discuss important innovations with conventional XAS, and compare the physical capabilities and beamline configuration for conventional XAS and HERFD-XAS experiments.

### Synchrotron radiation

Synchrotron radiation is generated from the relativistic acceleration of charged particles, such as electrons. This radiation is intense, collimated, and affords a continuous band of wavelengths spanning from the infrared (μm) to hard X-ray range (pm) [38,39]. Synchrotron-based X-ray techniques possess unique capabilities arising from the penetrating nature of the radiation and the ability to generate elemental signatures through the utilization of the characteristic binding energies of core electrons. Specifically, X-ray absorption fine structure (XAFS) spectroscopic techniques provide a unique atomic- and molecular-scale tool for studying the local structure surrounding an element of interest within a selected material or specimen [40]. This is a versatile technique and permits *in situ* investigation of the chemical form (or speciation) of an element in a range of samples including solutions, purified biomolecules (i.e. metalloproteins), and biological tissues. Although hyphenated chromatographic techniques, combining separation and detection methods, are often effective for speciation analysis, they require extensive sample preparation which may alter the native state of the analyte. This can be avoided, to some extent, with X-ray methods which require minimal sample preparation and hence are more likely to conserve the native analyte speciation of the sample.

Synchrotron X-ray methods inherently involve exposure of samples to high intensity ionizing radiation. As such, consideration must be given to the possibility of modification of elemental speciation and distribution of analytes during data collection and processing due to beam damage (photooxidation or photoreduction) [41]. This issue has been recognized for several decades, though recent advances in beamline equipment have improved the efficiency of data collection and procedures have since been established to improve maintenance of chemical integrity [6,42]. It is common practice to house samples in a cryostat at near liquid helium (He) temperature (4 K) which minimizes radiation damage and decreases the thermal motion of atoms, leading to improved spectra and informative content in XAS data [42]. Additionally, the XAS edge spectrum may be monitored for changes during data acquisition, and the sample may be repositioned relative to the beam between data-averaging scans to mitigate noted spectral alterations.

### Chemical insights from XAS spectra

#### Principles of X-ray Absorption Spectroscopy

**Figure 1.**
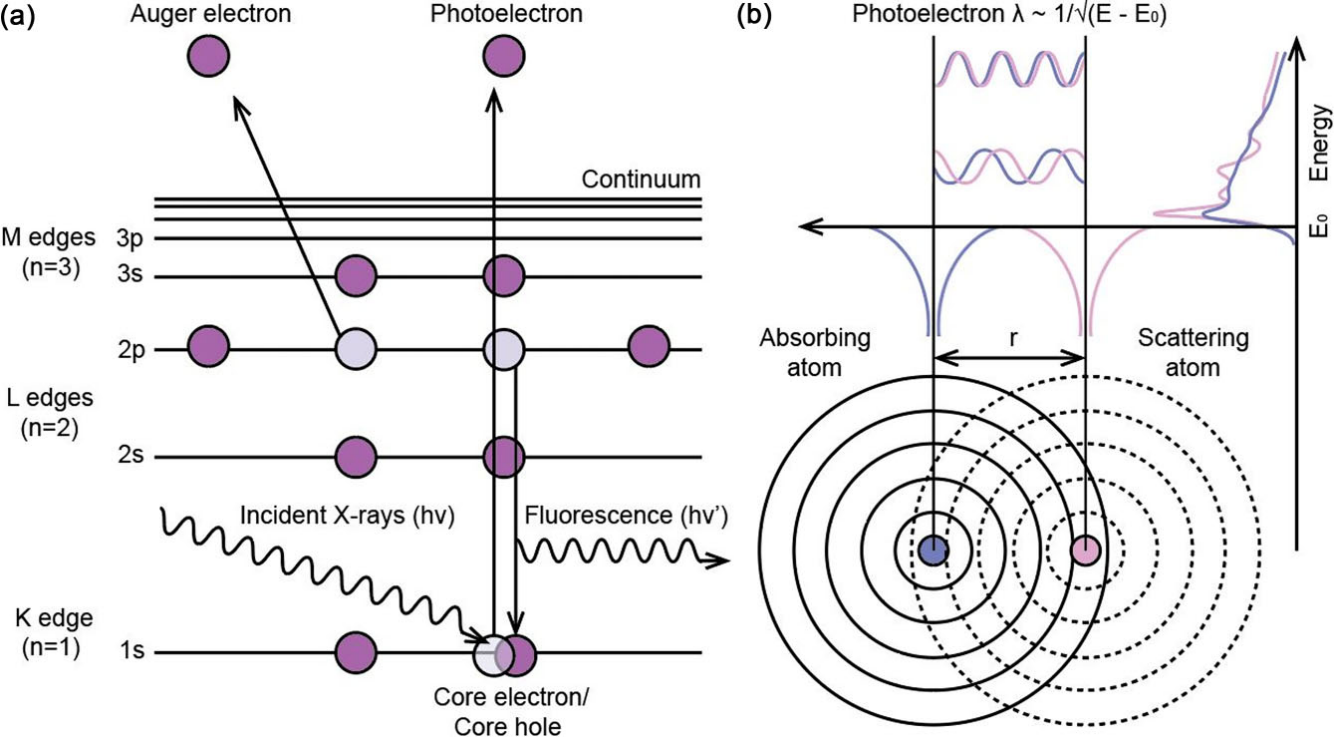
Principles of X-ray absorption spectroscopy. **(a)** the photoelectric effect, involving the absorption of an X-ray photon and the ejection of a core electron, creating a core-hole which is subsequently filled by an outer electron with concomitant emission of an X-ray fluorescent photon or an Auger electron. This is a simplified diagram; however, selection rules dictate that Auger emission of a 2*p* electron would be accompanied by the filling of the core-hole with an electron from the 2*s* shell (rather than 2*p*), **(b)** EXAFS produced due to scattering of the photoelectron from a neighboring atom. The scattered photoelectron can return to the absorbing atom resulting in modulation of the amplitude of the photoelectron wavefunction, subsequently modulating the absorption coefficient μ(E) and producing EXAFS oscillations. Note that the spherically symmetric waves depicted here represent a simplified view of the absorber and backscatterer interactions. for example, the final state de Broglie wavefunctions for both the outgoing and backscattered waves have an angular momentum $l$ = 1 for a K-edge absorption event.

Originating in the 1970s, XAS has become increasingly popular across a variety of scientific fields [43,44]. Incident X-rays from a synchrotron source are absorbed by matter via the photoelectric effect and, with sufficient energy, promote electrons from tightly bound core levels to the continuum (Fig. 1). The basic physical quantity measured is the X-ray absorption coefficient $\mu ( E )$ which describes the absorption of X-rays as a function of the X-ray energy $E$. A sudden increase in absorption corresponding to an ionisation event produces a sharp rise in the spectrum, commonly described as an ‘absorption edge’ [45]. Different edges are generated by targeting the excitation of electrons from different core levels, including K and L levels in the hard X-ray regime which probe XAFS with energies between 5 and 35 keV [40]. In XAS experiments with biological samples, this region includes the K- and L-absorption edges of elements from sulfur to high Z transition metals. The excited state generated by the primary photoexcitation event consists of an atom with a core-hole and a photoelectron. This core-hole is refilled within a fraction of a femtosecond (fs) by decay of an outer electron with concomitant emission of characteristic fluorescence energies or Auger emission [40]. Following an absorption event, the ejected photoelectron wave may be scattered by electrons in atoms surrounding the absorbing atom, creating quantum interference, which can be conceptualized as arising from constructive and destructive interference between outgoing and backscattered de Broglie photoelectron waves [44,45].

Relevant regions for analysis can be subdivided into (i) the X-ray absorption near-edge structure (XANES) spectra, collected within 100 eV of the absorption edge, reflecting the average coordination environment and oxidation state of an element, and (ii) the extended X-ray absorption fine structure (EXAFS) spectra, collected across a broader energy range, to estimate coordination number and bond lengths [40]. The data obtained from each region is complementary and can collectively provide electronic and structural information for the element of interest and the variety of species present, albeit with several limitations.

#### Near-edge spectra

Dipole-allowed transitions (i.e. Δ*l* ± 1) predominantly give rise to the features observed in near-edge spectra, therefore, the analysis of these spectra can yield information regarding the electronic structure of the compound, including the oxidation state and, in some cases, the geometry [43]. These near-edge or XANES spectra are often highly distinctive and can be used to determine mixtures of unknown chemical species present in biological samples. Through linear combination or least-squares fitting to a set of model compound spectra, the proportions of species containing the element of interest may be determined, though this method is restricted to identifying only chemical type rather than specific molecules (i.e. R—S—R, Hg(SR_2_)) with the identities of R-groups remaining potentially unclear. These fits are generally accompanied by principal component analysis (PCA) to help estimate the number of unique species in the multicomponent mixture [43,44,46]. Evidently, the analysis of XANES requires some prior anticipation of the chemical forms which are likely present in the chosen sample. Thus, the collection of XAS data for an extensive model compound library, which covers the breadth of relevant chemical species, can pose a limitation to studies in complex systems.

#### Extended X-ray absorption fine structure spectra

EXAFS oscillations arise from the interferences between the photoelectron waves of the absorbing atom and the neighboring atom or backscatterer, and the phase and amplitude of these oscillations are dependent on the backscatterer identity. Using this information, the backscatter can be distinguished, however, it is generally not possible to discriminate between backscattering atoms with similar atomic number [43]. Fitting of EXAFS data requires theoretical standards, computed via *ab initio* calculations in programs such as FEFF, or experimental standards derived from known reference compounds, collectively forming a library of model absorber-backscatterer pair phase and amplitude functions. The sum of the squares of the differences between experimental and calculated EXAFS is minimized in an iterative manner by adjusting the various structural parameters in the EXAFS equation (Equation 1),


(1) \begin{eqnarray*}
\chi \left( k \right) = \mathop \sum \limits_j \frac{{{N_j}{f_j}\left( k \right){e^{ - {R_j}/\lambda \left( k \right)}}{e^{ - 2{k^2}\sigma _j^2}}}}{{kR_j^2}}{\mathrm{sin}}\left[ {2k{R_j} + {\delta _j}\left( k \right)} \right]
\end{eqnarray*}

where $\chi ( k )$ is ‘the EXAFS’ or the oscillations as a function of the photoelectron wavenumber $k$ (in dimensions of 1/distance), *j* is an individual scattering path,$f( k )$ and $\delta ( k )$ are scattering properties of the neighboring atoms, $\lambda ( k )$ is the photoelectron mean-free-path, $N$ is the coordination number of neighboring atoms, $R$ is the distance to the neighboring atom, and ${\sigma ^2}$ is the mean-square deviation of the neighbor distance due to vibrational effects and static disorder [40,45]. Thus, given the scattering amplitude $f( k )$ and phase-shift $\delta ( k )$, and $\lambda ( k )$, the EXAFS equation permits the calculation of $N$, $R$ and ${\sigma ^2}$. Subsequently, this fit provides a structural characterization of the metal site ligation, including the type of neighboring atoms, the coordination number, and the bond distances [38,43].

Analysis of EXAFS data from biological samples poses a range of challenges, most notably (i) poor EXAFS data quality due to the low concentrations of probed elements and subsequent poor signal-to-noise ratio, and (ii) susceptibility to substantial residual errors resulting from the complexity of biological samples which contain mixtures of chemical forms of the element of interest. These issues cannot be eliminated but may be lessened by taking multiple scans to obtain higher quality EXAFS data, by utilizing an extensive model compound library, and by using XANES data to inform EXAFS fitting. Ice-diffraction from frozen microcrystalline water molecules in some samples can also make EXAFS measurement challenging by reducing the detector efficiency or necessitating longer data collection times. However, this can be minimized by rapid freezing in liquid nitrogen (LN_2_) cooled slurries (i.e. with isopentane), or by freezing with a glassing agent (i.e. glycerol or glycol) added to the sample where possible [44,47].

As the applications of XAS expand to increasingly complex systems, the limits of XANES and EXAFS analysis continue to be pushed. Elucidating chemical speciation via the use of either part of the XAS spectrum requires sufficient spectroscopic energy resolution. This presents a limitation for conventional XAS analysis of dilute biological samples given the core-hole lifetime and resulting peak broadening, which HERFD-XAS can alleviate with its enhanced energy resolution and background rejection. This provides a significant advantage over conventional XAS analysis, permitting application to a wider range of samples in metallomics research.

## Innovations with conventional XAS

In recent decades, researchers using XAS have made significant contributions to understanding the mechanisms that underpin the roles of metal ions in biological systems [6,48]. The utility of the technique extends across a range of scientific fields including physics, materials science, catalysis, biogeochemistry and environmental science. These applications are beyond the scope of our discussion and are covered extensively in existing reviews [49–52]. In this article, we focus on the discussion of innovations achieved using conventional XAS in metallomics research, emphasizing advantages over other techniques. In doing so, we will begin to describe the limitations of the technique before introducing how some of these obstacles can be overcome with HERFD-XAS.

### Understanding metalloenzymes

#### Rubredoxins

The practicality of XAS is exemplified in its contribution to the understanding of rubredoxins, Fe—S containing proteins which act to reduce superoxide species in some anaerobic bacteria and function as single-electron carriers in various biochemical pathways. The first protein crystal structure of rubredoxin, derived from *Clostridium pasteurianum*, was determined from 1.5 Å resolution X-ray diffraction data by Watenpaugh *et al*. in 1973. Their work reported a highly distorted Fe site with Fe—S distances in the range of 2.05 Å to 2.34 Å, potentially reflecting an entatic site [53]. However, this was inconsistent with results from the first Fe K-edge EXAFS analysis of rubredoxin from *Peptococcus aerogenes* in 1975—the pioneer EXAFS study in biological molecules—which indicated no distortion within the active site and reported an average Fe—S bond distance of 2.24 $ \pm \,\,$0.02 Å, with an average bond length increase of 0.05 Å upon reduction with dithionite [54]. A subsequent EXAFS study on *C. pasteurianum* rubredoxin in 1976, reported an average Fe—S bond length of 2.30 $ \pm \,\,$0.04 Å [55]. In following years, Watenpaugh and co-workers produced crystallographic data at a resolution of 1.2 Å which corroborates well with the Fe—S bond distances from EXAFS findings [56].

#### Nitrogenase: The FeMoco cofactor

Beginning in the late 1970s, EXAFS studies have played an integral role in our understanding of the active site structure of nitrogenases, a family of enzymes responsible for the N-fixing capabilities of diazotrophs. The enzyme is a heterodimeric (α_2_β_2_ tetramer) complex which serves to catalyze the ATP-dependent reduction of atmospheric dinitrogen gas (N_2_) to two molecules of ammonia (NH_3_), and is key to the bioavailability of one of life’s most essential elements [57]. The most widely studied nitrogenase group is the molybdenum-dependent (Mo-dependent) form. These nitrogenases exhibit substantially higher efficiency for N_2_ reduction, with a lower stoichiometric ratio of N_2_ to ATP (1:16) for conversion, relative to other nitrogenase varieties (i.e. Fe- and V-dependent nitrogenases, requiring 1:40 and 1:24 N_2_ to ATP, respectively) [58]. These Mo-dependent enzymes are composed of an Fe protein and a Mo—Fe protein, the latter of which contains a P-cluster and an elusive FeMo cofactor (FeMoco) [59].

The first Mo EXAFS derived structures, proposed in 1978, suggested sulfur bridged clusters potentially containing a linear Fe—Mo—Fe unit or a MoFe_3_ cubane structure [60,61]. Following the collection of Fe EXAFS data in 1982, this postulation was revised with experimental evidence pointing to a larger FeMoco cluster composed of multiple cubane units [62]. Unlike the previously discussed rubredoxin example, the crystal structure data collected for nitrogenase in *Azotobacter vinelandii* in 1992 was consistent with the early EXAFS experiments [63], with the X-ray spectroscopic data giving more reliable values for the Mo—S and Fe—S distances (∼2.35 Å), and Mo—Fe and Fe—Fe distances (∼2.7 Å) [60,63]. Alongside the investigation of nitrogenase, a similar debate regarding conclusions drawn from crystallographic data and EXAFS analysis arose in 1981 and 1982 surrounding the structure of [3Fe—4S] clusters in ferredoxins, described in a 1988 review by George and George [64].

Prior to these studies, EXAFS had been considered a secondary tool for crystallography, used to elucidate small corrections to existing models and ideas. Together, these examples are notable triumphs for XAS studies, highlighting the ability of XAS to yield results in biological solutions, a feat unachievable with crystallographic analysis which is explicitly limited to structural analysis of crystallized samples. In the analysis of solid crystal structures, X-ray diffraction (XRD) and XAS are better characterized as complementary X-ray methods, as the combined data from these two techniques is considered necessary to fully characterize the metal binding site of a metalloprotein [38,65]. EXAFS is also commonly used to determine structural details of metal binding sites at a higher bond-length accuracy (∼0.01 Å) than structures obtained by typical XRD (∼0.1-1 Å), with XANES providing the necessary information to determine the metal oxidation state and coordination geometry [38].

### Characterizing chelation drugs

More recently, the value of XAS has been illustrated in the development and rational design of chelator drugs, particularly for the purpose of mercury (Hg) chelation therapy. Among the most toxic heavy metal elements, Hg is a known perpetrator of health problems including neurological disease and myocardial infarction [66,67]. Clinical treatment of Hg poisoning requires binding of the heavy metal to a chelation therapeutic drug, formation of a chelate complex, and subsequent excretion. The binding efficacy of Hg(II) to the drugs meso-dimercaptosuccinic acid (DMSA) and dimercaptopropanesulfonic acid (DMPS) were investigated by George *et al*. in 2004 using a combination of Hg L_III_-edge XAS, chromatography and density functional theory (DFT). Analysis of EXAFS data collected from the titration of Hg(II) with DMSA and DMPS revealed no evidence that significant quantities of chelate complexes were formed in experiments [68]. These results prompted DFT explorations of the design of a custom chelator, optimized for diagonal coordination of Hg between thiol groups, using methylmercury mercaptide as a minimal model, with S atoms separated by twice the optimal Hg—S distance (2 $ \times $ 2.345 Å) [68]. Efforts were continued by Fu *et al*. in 2011, extending previous DFT calculations and deriving a revised optimum three-coordination geometry [Hg(SR)_3_]^−^, leading to a proposed benzene-1,3,5-triamidopropanethiolate ‘tripod’ chelator [69]. Further studies of the efficacy of new chelators such as this will inevitably require the use of EXAFS curve fitting analysis, demonstrating the importance of XAS as an *in situ* spectroscopic probe, providing chemically accurate descriptions of metals and their ligands in toxicology studies.

### Limitations of conventional XAS

#### The core-hole and lifetime broadening

Although the above examples illustrate the unique advantages of XAS, studies in recent years have exposed the shortcomings of this technique, emphasizing the need for development of advanced XAS methods. Conventional EXAFS is a valuable tool which provides the ability to extract local structural details around an absorber atom, yet it suffers from a notable lack of sensitivity for the detection and differentiation of light atoms in the surrounding structure. This limitation makes it essentially impossible to distinguish C, N, and O atoms within the proximity of a metal center and to discriminate R-groups between species with similar structural form, limiting interpretations to only the local environment surrounding the absorber atom [70]. In general, significant structural and electronic information can be gained from the XANES region and pre-edge signal of transition metals. This is most pronounced for first row transition metals which generate pre-edge peaks from 1*s*$ \to $ 3*d* transitions containing information about the lowest unoccupied molecular orbital (LUMO) and reflecting the geometric and electronic structure [71]. However, the core-hole lifetime broadening of the 1*s* electron hole smears out essential features of the pre-peak, limiting the information which can be extracted from the conventional XANES spectra. Combined with the influence of undesirable background inelastic scatter on poor signal-to-noise ratio in the EXAFS of dilute samples, the quality and reliability of XAS analysis is inhibited in many biologically relevant samples.

The ‘core-hole’ refers to the vacancy generated in the core orbital due to excitation of a photoelectron, thus, an atom with a core-hole is in an excited state. This state has a finite lifetime and will decay rapidly as electrons in higher energy states fill the core-hole, simultaneously emitting a fluorescent photon or generating non-radiative decay products. The Heisenberg uncertainty relation (Equation 2),


(2) \begin{eqnarray*}
{\mathrm{\Delta }}{E_L}{\mathrm{\Delta }}t \mathbin{\lower.3ex\hbox{$\buildrel>\over {\smash{\scriptstyle\sim}\vphantom{_x}}$}} \hbar /2
\end{eqnarray*}

describes the association between the core-hole lifetime ${\mathrm{\Delta }}t$ and the lifetime broadening of spectroscopic features associated with the primary photoexcitation, ${\mathrm{\Delta }}{E_L}$, which has a finite energy width [45]. For a given photoexcitation (*e.g*. of the 1*s* level to give a K-edge spectrum), the core-hole lifetime decreases with increasing absorber atomic number (Z), and the lifetime broadening increases according to Equation 2. Lifetime broadening introduces a Lorentzian broadening to transitions comprising the XANES, which in turn serves to obscure much of the detail in the spectra, especially for heavier elements, limiting the utility of XAS for many elements of interest in metallomics research.

#### The XAS beamline configuration

**Figure 2.**
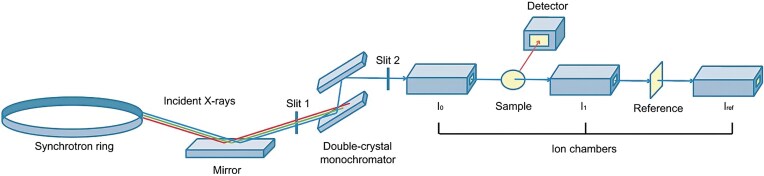
Generalised schematic of instrument configuration for a conventional XAS experiment using synchrotron radiation. A focusing device (e.g. K-B mirrors or toroid mirror) has not been included in this simplified diagram, however, this component is generally positioned after the monochromator.

The spectroscopic resolution of an XAS experiment is inevitably influenced by the resolution of the beamline X-ray optics. One typical arrangement of optics and instrumentation for a conventional XAS experiment is depicted in Fig. 2. In this set-up a monochromator is required to select and vary the incident energy across the threshold energy (${E_0}$) of the element of interest according to Bragg law (Equation 3),


(3) \begin{eqnarray*}
n\lambda = 2{d_{hkl}}sin\theta
\end{eqnarray*}

where ${d_{hkl}}$ is the spacing between diffracting lattice planes, $\theta $ is the angle of reflection, and $\lambda $ is the selected wavelength which can then be related to the photon energy [38]. XAS experiments commonly utilize a double crystal monochromator, although four-crystal monochromators can be used to reduce the intensity of tails in the monochromator resolution function (Gaussian broadening), with the compromise of additional expense and complexity in alignment, and an inevitable loss of X-ray intensity [45]. The energy of the beam emerging from the monochromator is adjusted by changing the angle of incidence onto the crystal. Silicon monochromators (i.e. Si(111), Si(220), or Si(311)) are typically used for energies above 2 keV, due to their desirable thermal properties and high quality crystals, cut along a specific Millar plane to ensure precise diffraction energies according to the Bragg condition [45]. Each crystal cut has an inherent energy resolution due to the finite angular width over which Bragg diffraction occurs, called the Darwin width, and this provides a physical limitation on the resolution of the monochromator. In modern high-intensity beamlines the power densities on the first crystal from the incident polychromatic ‘white’ beam can be as high as 1-10 W/mm^2^ and pressurized LN_2_ is used to remove heat, but even so there may be a thermal bump where the beam strikes the first crystal, which will degrade the energy resolution. In high-intensity beamlines monochromator crystals are typically maintained in a vacuum, or in the case of low-intensity beamlines, a He atmosphere may be used. To optimize data quality, the ideal monochromator resolution width should be several times smaller than the intrinsic lifetime broadening of the absorption edge [45].

The typical beamline configuration also employs a focusing device to collimate the beam which is generally positioned directly after the monochromator and before the first ion channel. These focusing mirrors (one or more) may be flat, bent-flat, or toroidal [45]. Kirkpatrick-Baez (K-B) mirrors, consisting of two elliptically bent mirrors positioned side-by-side and perpendicular (one horizontal and one vertical) with a common X-ray focal point, are widely utilized for generating a small and focused X-ray beam in XAS experiments, as these reflective optics provide the advantage of tunable focal distances [72]. Alongside the focusing device, slits at several locations in the beam pathway are utilized to control the physical width of the beam and its angular speed [45].

When using XAS for the measurement of concentrated samples, the linear absorption coefficient $\mu ( E )$, which varies with sample thickness ($x$), is determined according to the Beer-Lambert law (Equation 4),


(4) \begin{eqnarray*}
\mu \left( E \right)x = {\mathrm{ln}}\left( {{I_0}/{I_1}} \right)
\end{eqnarray*}

where ${I_0}$ and ${I_1}$ are the incident and transmitted X-ray flux intensities, commonly measured using ion chambers. Variations of $\mu ( E )$ are measured by scanning the incident energy beam $E$ [38]. However, the typically low metal concentrations of biological samples (below ∼2 mM) result in weak absorption in transmission mode, hence, data collection is carried out in fluorescence mode and recorded by an X-ray detector positioned at 90^o^ to the incoming beam [38]. The detector absorbs the incoming radiation, after being either scattered or emitted as fluorescence from the sample, with the latter containing the signal of interest. The absorption by the detector generates photoionization of the atoms within the detector and a cascade of excitations which are converted to electrical signals. These detectors typically operate in two modes; (i) pulse-counting, where each absorbed photon generates a pulse of charge via the separation of electrons and holes, and (ii) current integration, which does not distinguish the energies of photons but simply determines the rate of absorbed energy from the beam [45].

Conventional XAS typically utilizes solid-state pulse-counting detectors containing multiple silicon (Si) or germanium (Ge) elements, typically ≥ 13 elements, however some commercial drift detectors contain as few as 4 elements. Ge array detectors are often selected as the higher Z permits improved absorption, translating to better efficiency, with high-energy photons (above 15 keV) [43,45]. Whilst electronic deadtime effects limit the maximum count rate per array element to around a few hundred thousand counts per second (depending on the detector system), multi-element detectors can achieve millions of counts per second. However, a trade-off exists between the maximum count rate and energy resolution due to time constraints of the electronics. Notwithstanding this, the best possible energy resolution from these semiconductor detectors is typically no better than 130 eV [45]. This is sufficient for most purposes but can present issues for the analysis of dilute biological samples. At higher flux, the background signal from scattered radiation increases and the detector count-rate capability can be overwhelmed [73]. Furthermore, detector non-linearities can arise due to inadequacies in readout electronics and background artefacts resulting from inefficient charge collection and the effects of inelastic (Compton) scattering in the energy region of interest. Limited background rejection and related issues can ultimately result in signal averaging with higher-than-predicted noise levels based on photon-statistics, reducing speciation sensitivity in dilute and ultra-dilute biological samples [74]. Accompanying the low signal-to-noise ratio, EXAFS oscillations for coordinated metal centers experience a damping effect due to the structural disorder existing in the coordination sphere, generally limiting the extension of measurable EXAFS data to 10-14 Å^−1^ in $k$ space [75].

## The new capabilities of HERFD-XAS

Although XAS offers the convenience of minimal sample preparation and the preservation of the native chemical state, the core-hole lifetime broadening effects and poor signal-to-noise ratio collectively limit the ability to produce reliable speciation data in complex and highly dilute biological samples [38,75]. Fortunately, the rise in availability of high brilliance synchrotron radiation facilities in recent decades has provided opportunities to develop high-resolution X-ray spectroscopic techniques, establishing X-ray emission spectroscopy (XES) and resonant inelastic X-ray scattering (RIXS), alongside HERFD-XAS, as advanced characterization tools. Here we describe the experimental design of HERFD-XAS and discuss the utilization of this emerging high energy resolution method for the purposes of metallomics research, highlighting improvements demonstrated in speciation analysis of mercury (Hg) and selenium (Se).

### Obtaining enhanced resolution and background rejection

#### Minimizing lifetime broadening

The combined Lorentzian and Gaussian broadening effects, from core-hole lifetime and beamline optics, respectively, lead to an overall Voigt peak shape in conventional XAS spectra [45]. To provide relief from the core-hole lifetime broadening, HERFD-XAS measures X-ray fluorescence with significantly higher energy resolution than the natural emission linewidth. As previously described, the interactions between the X-ray and sample can be described in two stages: (i) absorption of an X-ray photon by excitation of a core-electron generating a photoelectron and a core-hole, and (ii) filling of the core-hole by decay of an electron from a higher energy bound state via emission of an X-ray fluorescent photon with creation of a secondary core-hole. This secondary core-hole has a notably longer lifetime than that of the core-hole derived from the initial excitation.

For example, the Se Kα_1_ fluorescence line derives from a 2*p*_3/2_→1*s* transition; the 1*s* core hole has a lifetime $\Delta t$ of approximately ¼ fs, which (from Equation 2) gives a lifetime broadening ${\mathrm{\Delta }}{E_L}$ of ∼3 eV, while the 2*p*_3/2_ core-hole that results from the Se Kα_1_ fluorescence emission has $\Delta t$ closer to 1 fs, and a correspondingly smaller ${\mathrm{\Delta }}{E_L}$. By measuring the fluorescence with much better energy resolution than the natural linewidth, the spectroscopic resolution becomes more dependent on the lifetime of the core-hole created by the fluorescent event. Consequently, the peak-shapes of HERFD-XAS are often more Gaussian, while those of conventional XAS are more Lorentzian, due to the greater importance and influence of the resolution of the beamline. Thus, HERFD-XAS measurements target the fluorescence emission stage, hence the incorporation of ‘fluorescence detected’ into the title of this methodology.

A notable caveat of the HERFD-XAS method is that good energy resolution of the spectrometer measuring fluorescence is crucial. If the resolution is inadequate, then enhanced spectroscopic resolution may not be achieved. It is also important to note that HERFD-XAS is not immune to the effects of fluorescence self-absorption, which is well recognized in conventional XAS experiments [76–78]. This typically occurs for thicker and more concentrated samples which present a non-linear relationship between X-ray fluorescence and the absorption coefficient $\mu ( E )$ [78,79]. Fortunately, the fluorescence self-absorption distortion in HERFD-XAS can be corrected mathematically, however, unlike the straightforward corrections applied to conventional fluorescence XAS, this requires an independent measurement of the absorption coefficient at both the incident beam energy and the fluorescence energy. Fortunately, this self-absorption effect is not generally relevant to biological samples which are typically thinner materials and/or contain the absorbing element in dilute or ultra-dilute concentrations [78,79].

#### X-ray filter systems

Although conventional XAS methods can achieve detection of XAFS at concentrations approaching parts-per-million (ppm) level dilution, improvements to this limit may evolve from beamline development, and the design and implementation of innovative detector systems capable of better isolating the fluorescence line of the element of interest [73]. The desired fluorescence signal is typically muddled by the influence of background arising from elastic and inelastic scattering. The three types of detectors commonly utilized in XAFS experiments, either in isolation or combination, are (i) multi-element solid-state detectors, (ii) filter-silt systems, and (iii) crystal analyzers [73,80]. As we previously described, solid-state Si and Ge detectors are limited by their maximum count rate, though this weakness is typically mitigated using several detector elements in unison [73]. However, these detectors may still be overwhelmed by high flux beams and are susceptible to background scattering leaking into the fluorescence channel [73].

Fortunately, the selectivity of solid-state detectors can be easily improved by utilizing X-ray filter assemblies to strategically remove background scattering before the final detector is reached. X-ray filters can be derived from metal (Z) foils which act to selectively absorb elastic and inelastic scattered X-rays for incident energies above the K-edge of the subsequent (Z + 1) absorber atom. However, this is a general rule for K-edges and does not apply to L-edges. For example, the best filter for the Hg L_III_-edge is gallium (Ga). Additionally, placing a Soller-type slit assembly between the filter and detector minimizes the influence of fluorescence or secondary scatter from the filter on the total count rate capability of the detector [80,81].

#### Probing the RIXS plane

**Figure 3.**
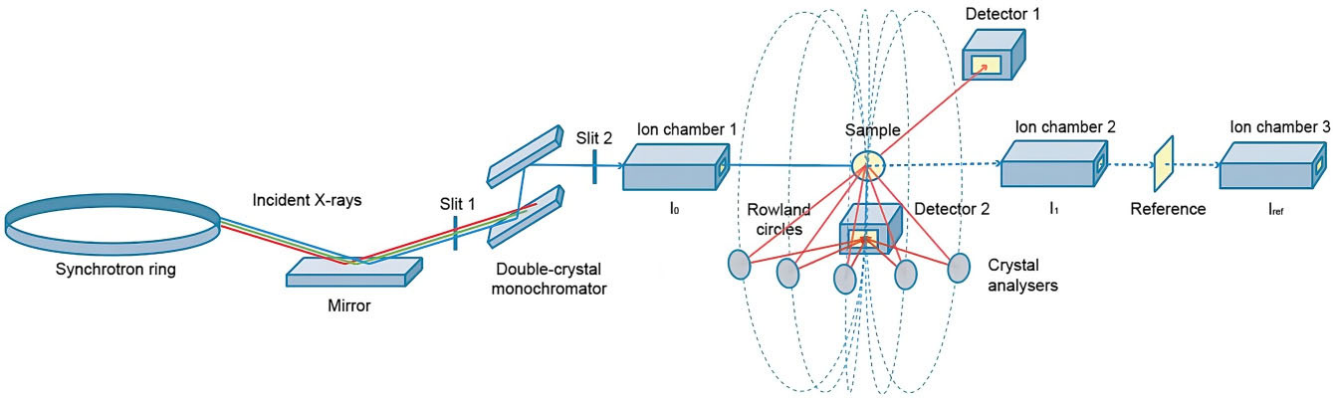
Generalised schematic of instrument configuration for a HERFD-XAS experiment using synchrotron radiation. The X-ray detector (Detector 2) is aligned on a Rowland circle with the sample and typically positioned below the sample (within a He cryostat). Note that the Rowland circles are all equivalent in size. The number and type of crystal analysers and the specific geometry of the arrangement is variable between experimental designs. A focusing device (e.g. K-B mirrors or toroid mirror) has not been included in this simplified diagram, however, this component is generally positioned after the monochromator.

The advanced detector set-ups for HERFD-XAS typically employ a crystal analyzer system, shown schematically in Fig. 3, to eliminate the inelastic (Compton) X-ray scattering which often dominates background signal in conventional XAS detectors. Inelastic scatter at energies close to the absorption edge creates resonant effects which strongly influence scatter intensity and may be interpreted to provide structural information about the scatterer [45]. Conventional fluorescence XAS experiments measure the sum of all transitions between the higher occupied levels and the core level, whereas HERFD-XAS probes the resonant inelastic X-ray scattering (RIXS) plane to observe a smaller subset of transitions central to the peak. The RIXS plane can be recorded by scanning both the incident energy and the analyzer while monitoring the fluorescence intensity. To clarify this nomenclature; ‘resonant’ refers to the fact that the incident energy is scanned through transitions in the absorption edge, and ‘inelastic’ indicates that there are often energy losses.

**Figure 4.**
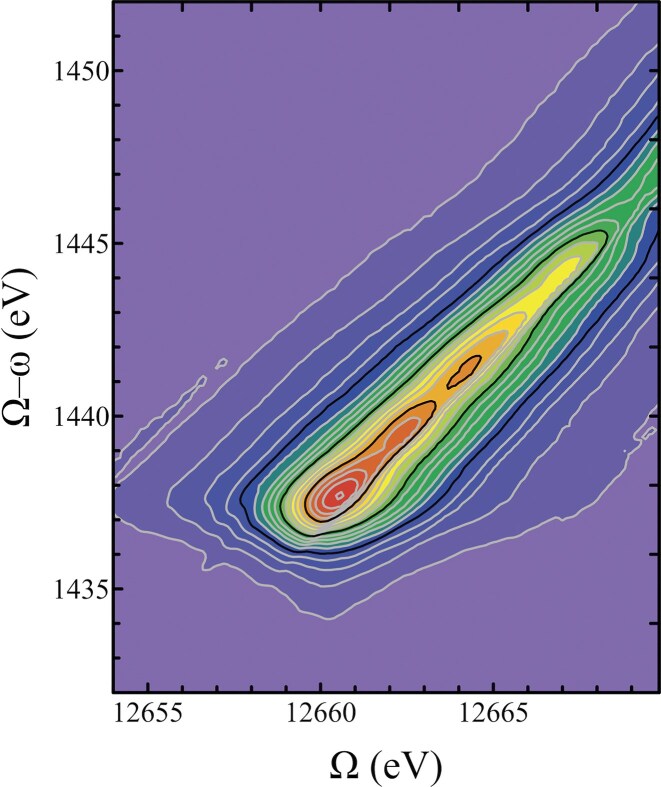
RIXS plot depicting the Se Kα_1_ RIXS plane for ZnSe_(s)_. The plot shows the intensity of emitted X-rays plotted as a function of incident X-ray energy (Ω, abscissa) and the energy transfer computed as the difference between incident and emitted X-ray energies (Ω-ω, ordinate). The contours are shown from purple (low) to red (high), and a diagonal cut across the plane intersecting the peaks would be selected for the collection of HERFD-XAS.

RIXS is an element-selective technique which interrogates electronic excited states using X-ray radiation, providing specific information about the occupied and unoccupied orbitals with contributions from the probed atom, and represents a two-electron process involving (i) the promotion of a core electron to an unoccupied orbital above the Fermi level via the absorption of a photon, and (ii) subsequent filling of the core vacancy by an electron from a higher-lying energy level, accompanied by the emission of a fluorescent photon [82,83]. Thus, the information gained about the electronic state energies can be used to inform the selection of a specific fluorescence line for HERFD-XAS experiments. The RIXS plane is represented as a surface or contour plot of the incident energy (${E_{in}}$ or ${\mathrm{\Omega }}$) and the final state energy (${\mathrm{\Omega }} - \omega $, where $\omega $ is the emitted energy), as exemplified for ZnSe(s) in Fig. 4. The crystal analyzer can be set to the emitted energy and scanned across the incident energy (a constant emission energy scan) to generate a diagonal cut across the RIXS plane. Combined with deconvolution analysis, scanning through the RIXS plane can be used to effectively minimize the line broadening of the spectrum [84]. To reliably compare standards and sample HERFD-XAS spectra, the energy window must be identical for the selected fluorescence peak, as the measured edge position in a HERFD-XAS scan is dependent on the chosen emitted energy ($\omega $) [85].

#### Crystal analyzers

High-resolution spectrometers, such as those designed for HERFD-XAS, commonly utilize Bragg-type bent crystals to permit efficient collection of photons, capturing a large solid angle for diffraction of scatter or fluorescence back to a common spot on the detector [80,86]. These arrangements can have different geometries and may be separated into two modes, (i) dispersive mode which is set up with Von Hamos geometry and utilizes a cylindrical bending crystal to focus fluorescence photons from the sample onto a position-sensitive detector, and (ii) point-to-point mode, with Johann or Johansson geometry, which utilizes spherically curved crystals to focus the fluorescence onto the detector [86–88]. A further variation is provided by what is known as the inside-Rowlands geometry, which can be used with Johansson geometry crystals, which gives an energy dispersive readout on a position-sensitive detector.

Johann geometry, considered the most common analyzer system with hard X-ray studies, gives a good compromise of ease of construction with high energy resolution. A Johann array consists of multiple spherically bent crystals each arranged on a Rowland circle of radius $r$, with a bend radius of $2r$ for each concave-curved diffracting crystal analyzer. An important aspect of the Johann spectrometer is that near-90° Bragg diffraction is required for the best analyzer energy resolution; at smaller angles the spectroscopic resolution starts to become very sensitive to the X-ray spot size on the sample (the source, for the analyzer). This means that analyzer crystals are often specific to an element. For example, the best choice for Se Kα_1_ HERFD-XAS is Si(844) with a Bragg angle (θ_B_) of 85°, whereas use of a Ge(844) would give θ_B_ = 73°, and the experiment would show a significantly poorer resolution enhancement above conventional XAS. Indeed, there are published examples of HERFD-XAS using a poor choice of analyzer crystal, demonstrating limited improvements in spectroscopic energy resolution relative to conventional XAS.

#### Sample preparation and analysis

Preparation of samples for HERFD-XAS experiments involves similar protocols as those applied to conventional XAS. Crucially, since these XAS techniques offer elemental selectively and sensitivity to oxidation state and coordination environment, sample preparation prioritizes minimal pre-treatment, avoiding destructive extraction and digestion protocols and favoring preservation of the native chemistry of the analyte. Solids including powders, lyophilized cell pellets, and biological tissues, may be directly packed into plastic cuvettes enclosed with metal-free polyimide adhesive tape and presented to the beam. As previously mentioned, solid samples must be dilute, ideally containing ≤ 0.5 wt% of the analyte of interest, to mitigate the effects of fluorescence self-absorption [78,79]. Similarly, solutions containing model compounds and biological lysates/liquids may be loaded into lucite or Delrin cuvettes and sealed with metal-free adhesive tape. However, unlike conventional XAS, the enhanced energy discrimination of HERFD-XAS provides relief from X-ray diffraction issues arising from ice-crystal formation during liquid sample freezing, hence, addition of glycerol or other glassing agents is not required.

**Figure 5.**
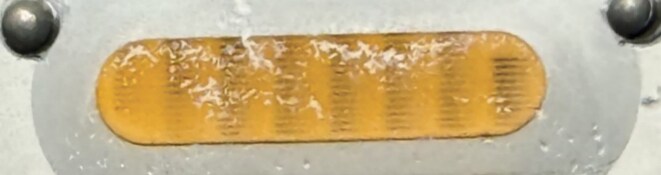
Radiation-induced color centers in a 1 wt % sample of GdPO_4_.0.5H_2_O in boron nitride following Gd Lα_1_ RIXS measurements at cryogenic temperature. The dark horizontal streaks in the sample correspond to regions exposed to the X-ray beam. The sample window is ∼20 mm wide in this image.

As described above for conventional XAS experiments, radiation hardness testing is equally essential for HERFD-XAS measurements, as photodamage to the sample from repeated exposure to the X-ray beam abolishes valuable information about the original speciation of the analyte of interest. This is highlighted in Fig. 5 which depicts visually discernable X-ray generated color centers on a gadolinium phosphate (GdPO_4_.0.5H_2_O) standard following Gd Lα_1_ RIXS measurements at multiple spots across the sample, illustrating the presence of potential radiation damage. As a simple strategy, radiation damage can be evaluated by sampling two sweeps per spot on the sample and comparing the peak positions and intensities at the near-edge energies between the two consecutive spectra. Taken together, data collection at cryogenic temperatures (∼10 K) and sequential shifting of the sample to irradiate a new unexposed spot for each measurement, can effectively minimize the impact of sample photodamage.

Existing software packages for conventional XAS data analysis, such as EXAFSPAK, can be utilized for data reduction, background subtraction, processing, and fitting of HERFD-XAS data. Relevant guides for these software packages are readily accessible online. However, linear combination fitting (LCF) of HERFD-XAS spectra using model compound spectra recorded at individually optimized emission energies (as informed by the RIXS) requires additional consideration, as appropriate corrections for fluorescence line shifts must be applied if fitting samples containing a mixture of formal oxidation states. A rigorous fluorescence shift correction method for LCF analysis using EXAFSPAK has been described in recent work by James *et al*. for the interpretation of Hg Lα_1_ HERFD-XAS spectra from biological tissues [89].

### Direct comparisons of conventional XAS and HERFD-XAS

#### Selenium speciation

Unique experimental designs have been put into practice in several recent studies, often aiming to scrutinize the strengths and limitations of HERFD-XAS for specific elements of interest. This comprehensive approach is seen in the work published by Nehzati *et al*. in 2021, examining HERFD-XAS as an advanced analytical method for Se speciation [90]. An enigmatic and perhaps underappreciated essential element of life, Se is the heaviest non-halogen *p*-block element with an established role in biological systems. It is incorporated into proteins as a selenocysteine residue furnishing the selenoproteome, a set of enzymes that facilitate cellular redox maintenance and thyroid regulation [91]. Although essential for human health, Se exhibits a uniquely narrow optimal dose range with physiological levels outside of this range associated with various diseases including neurodegenerative disorders, cardiovascular malfunction, diabetes, infertility, and some forms of cancer [91,92]. The parabolic relationship between Se status and disease risk is further complicated by its observed highly speciation-dependent biological activities [92], and an accompanying limited understanding of the metabolic routes of various dietary and non-dietary selenospecies. In addition, Se exhibits complex antagonistic and/or synergistic relationships with toxic elements, including Hg [93–95] and arsenic (As) [96–98], whose biological impacts we also recognize as highly dependent on speciation, although their precise mechanisms of action remain elusive.

The low abundance of Se presents a major challenge for studies of this element in biological systems, with typical concentrations in organs and tissues in the low μM range [99], approaching the practical detection limit for conventional XAS. In addition, as we have discussed, conventional XAS offers poor spectroscopic resolution in the hard X-ray regime, showing substantial broadening due to short core-hole lifetime, which is ∼1.41 $ \times $ 10^−16^ s for the Se K-edge at ∼12 658 eV [100]. Dramatic improvement in speciation sensitivity has been observed by Nehzati *et al*., for Se Kα_1_ HERFD-XAS spectra relative to conventional Se K-edge XAS. In this analysis, HERFD-XAS observes a subset of 2*p*_3/2_$ \to $ 1*s* transitions which give rise to the Kα_1_ fluorescence line, the most intense fluorescence line for Se [101,102].

The HERFD-XAS analysis reported in the Nehzati study was conducted at the Stanford Synchrotron Radiation Lightsource (SSRL) on the SPEAR3 storage ring on beamline 6-2, which utilizes a Si(311) double-crystal monochromator with ∼0.4 eV incident X-ray resolution. The Kα_1_ emission line was recorded using an array of seven Si(844) crystal analyzers in the Johann geometry, with an energy resolution of 0.95 eV, and output registered by a single-element Si drift Vortex detector [90]. The Se Kα_1_ HERFD-XAS spectra were compared to conventional Se K-edge XAS measured at SSRL on SPEAR3 with a Si(220) double-crystal monochromator (∼0.8 keV resolution) and a 30-element Ge detector, employing silver Soller slits and As filters for controlled maintenance of detector rates. For both methods, Se-containing samples were prepared as finely ground solids, mixed with a boron nitride diluent to < 1 wt % Se, and packed into 1 mm thick aluminum plates sealed with polyimide adhesive tape [90].

**Figure 6.**
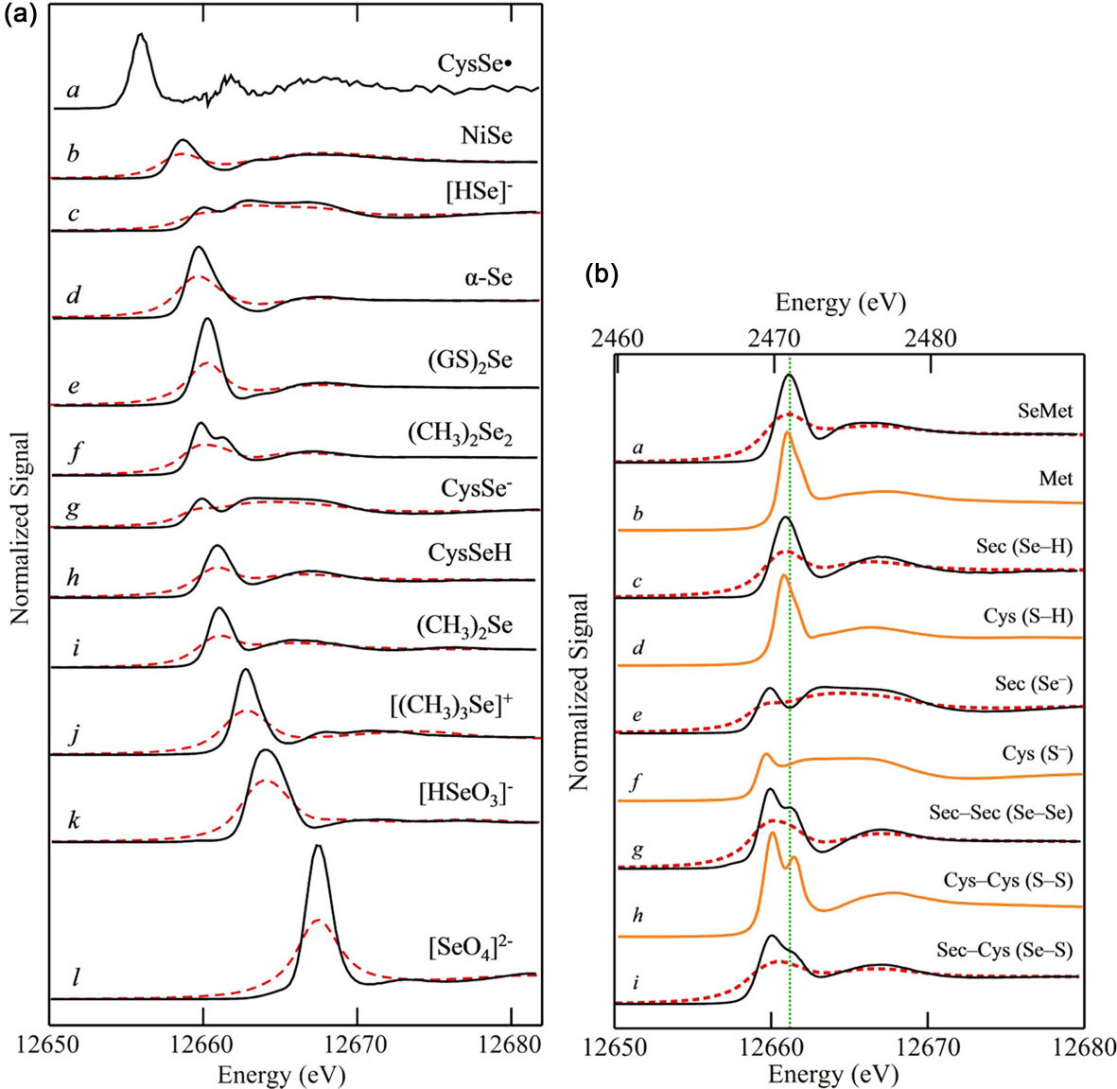
Conventional XAS and HERFD-XAS spectra collected for environmentally and biologically relevant Se compounds. **(a)** Chemical speciation sensitivity of Se Kα_1_ HERFD-XAS (black lines) relative to conventional Se K-edge XAS (red dashed lines) of the same aqueous solutions (1 mM Se, pH 7.4) with the following exceptions: a, lacking XAS; b, powder; d, α-Se suspension precipitated from 1 mM sodium selenite reacted with 10 mM reduced glutathione; h, pH 1.0. Cys, cysteine; GS, glutathione. **(b)** Comparisons of Se Kα_1_ HERFD-XAS (black lines) and conventional Se K-edge XAS (red dashed lines) of seleno-amino acids (lower abscissa), and S K-edge XAS of S congeners (orange lines; upper abscissa) for aqueous solutions (1 mM Se, 60 mM S, pH 7.4) with the following exceptions: c, pH 1.0; f, pH 10; h, oxidized glutathione solution due to limited solubility of cystine. Offset between abscissas aligns selenomethionine and methionine maxima. Met, methionine; Sec, selenocysteine; coordination in parentheses. The broken green line emphasizes variations in peak positions. [Reproduced with permission from Ref. 90; Nehzati, S, Dolgova, NV, James, AK, *et al*. High energy resolution fluorescence detected X-ray absorption apectroscopy: An analytical method for selenium speciation, *Anal Chem*, 2021;**93**: 9235-43. © 2021 American Chemical Society].

The overlays of collected Se Kα_1_ HERFD-XAS spectra and conventional Se K-edge XAS spectra (Fig. 6a) for a selection of environmentally and nutritionally relevant Se standards demonstrate meaningful improvements in spectroscopic resolution and enhanced speciation sensitivity of HERFD-XAS. Notable improvement was observed for aqueous selenate [SeO_4_]^−2^, with the HERFD-XAS spectrum demonstrating a ∼2-fold increase in resolution for the major peak and comparative full width half-maxima of 1.98 eV for HERFD-XAS and 3.79 eV for conventional XAS. Similar enhanced spectroscopic detail was observed in the Se Kα_1_ HERFD-XAS spectra of biologically relevant seleno-amino acids (Fig. 6b), with resolution approaching that of S congeners [90].

**Figure 7.**
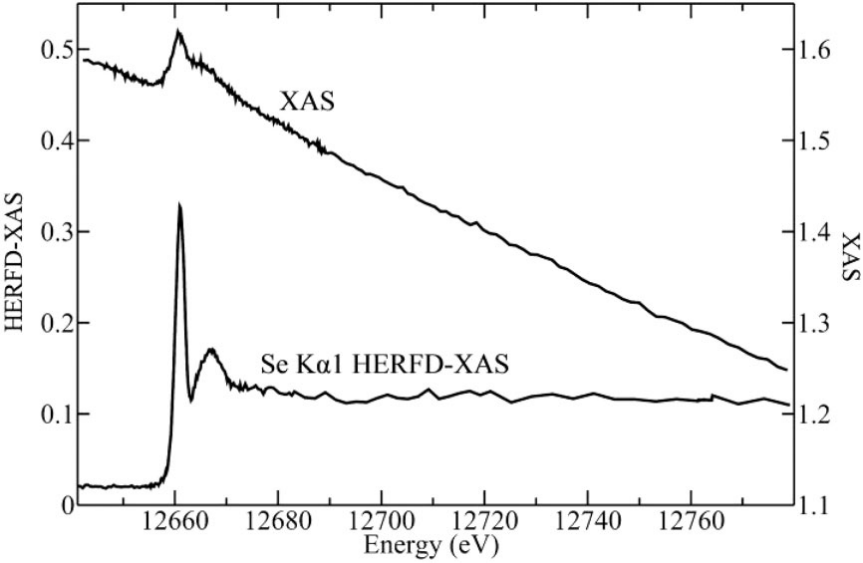
Se K-edge XAS (right ordinate) and Se Kα_1_ HERFD-XAS (left ordinate) of *Lutjanus peru* skeletal muscle at 10 K, containing ∼5 μM Se. Data was acquired on the SSRL SPEAR3 storage ring on beamlines 9-3 (XAS) and 6-2 (HERFD-XAS) delivering ∼2 × 10^12^ and ∼4 × 10^12^ photons per second, respectively. Data acquisition time was 35 min for each spectrum. [Reproduced with permission from ref. 90; Nehzati, S, Dolgova, NV, James, AK, *et al*. High energy resolution fluorescence detected X-ray absorption spectroscopy: an analytical method for selenium speciation, *Anal Chem*, 2021;**93**: 9235-43. American Chemical Society].

These high-energy-resolution spectra permit the detection of a subtle shift to lower energy between selenocysteine (SeCys) and selenomethionine (SeMet), which is observed in the spectra of S-containing analogues and enables discrimination between these residues with linear combination analyses. Analysis of red snapper (*Lutjanus peru*) skeletal muscle containing ∼5 μM Se revealed a significant improvement in signal-to-noise ratio with HERFD-XAS compared to conventional XAS, with equivalent acquisition time but a ∼42-fold lower solid angle for the analyzer array compared to the XAS detector. In contrast to the high degree of sloping background observed in the Se K-edge spectra, the Se Kα_1_ HERFD-XAS spectra shows negligible background and high energy selectivity (Fig. 7) [90], demonstrating a substantial improvement in spectroscopic resolution for Se in dilute biological samples.

#### Mercury speciation

In 2022, Nehzati and co-workers investigated the utility of Lα_1_ HERFD-XAS as an approach for the determination of the chemical speciation of Hg, with quantitative comparisons to conventional Hg L_III_-edge XAS [74]. As we have previously introduced, Hg has no known essential role in living organisms, but rather it is renowned for its toxicity, with organometallic Hg compounds posing a particular threat to the central nervous system of vertebrates [103–105]. Using HERFD-XAS to probe the RIXS plane, the Hg Lα_1_ peak and a subset of 3*d*_3/5_$ \to $ 2*p*_3/2_ fluorescence emission transitions were selected to essentially eliminate both core-hole and exchange broadening, and therefore substantially enhance spectroscopic resolution.

In this study, HERFD-XAS spectra were collected at the same location and beamline as the Se Kα_1_ HERFD-XAS spectra obtained by Nehzati and co-workers in 2021 [90], using a Si(311) double-crystal monochromator, an upstream Rh-coated mirror for harmonic rejection (∼18 keV cut-off energy), and downstream Si drift detector. An array of seven spherically bent Si(555) crystal analyzers in Johann geometry were selected to measure Hg Lα_1_ emission [74].

**Figure 8.**
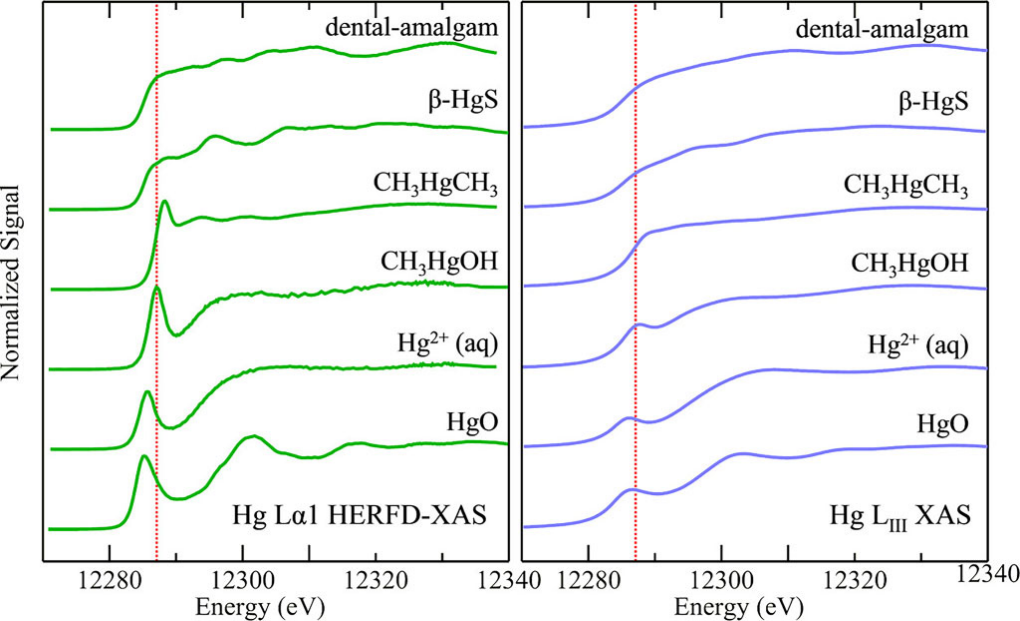
Comparison of Hg Lα_1_ HERFD-XAS (left panel, green lines) with conventional Hg L_III_ XAS (right panel, blue lines) for selected Hg standard compounds. The vertical broken red lines, at the same energy in the two panels, emphasise small displacements in the spectral features. Spectra were recorded for solid samples of dental amalgam, β-HgS, and HgO, while CH_3_HgCH_3_ was incorporated with a 1 mM isopropanol solution, and CH_3_HgOH and Hg^2+^(aq) were prepared as 1 mM aqueous solutions of methylmercury hydroxide and mercuric acetate, respectively. Data was acquired on the SSRL SPEAR3 storage ring on beamlines 7-3 (XAS) and 6-2 (HERFD-XAS). [Reproduced with permission from Ref. 74; Nehzati, S, Dolgova, NV, Young, CG, *et al*. Mercury Lα1 High energy resolution fluorescence detected X-ray absorption spectroscopy: a versatile speciation probe for mercury, *Inorg Chem*, 2022;**61**: 5201-14. © 2021 American Chemical Society].

Conventional Hg L_III_-edge XAS and Hg Lα_1_ HERFD-XAS spectra were compared for a range of Hg species, including mercury oxide (HgO), methylmercury hydroxide (CH_3_HgOH) and aqueous Hg^2+^, representing different coordination environments. Analogous to the improvements observed in Se Kα_1_ spectra, a substantial enhancement in resolution can been seen (Fig. 8) in the Hg Lα_1_ HERFD-XAS spectra relative to conventional XAS. Hg Lα_1_ HERFD-XAS spectra collected for mercury chlorides, HgCl_2_ and [HgCl_4_]^2−^, were found to exhibit excellent agreement with simulated spectra using time-dependent and restricted open shell configuration interaction singles DFT methods (TD-DFT and DFT/ROCIS, respectively). Additionally, Hg Lα_1_ HERFD-XAS spectra collected for a range of organometallic mercury compounds were found to appear broadly similar to each other, however sufficient unique detail in peak structure and position was present to permit discrimination between spectra from individual methylmercury compounds. Importantly, the authors emphasize that a good signal-to-noise ratio is necessary to distinguish between these species with confidence [74]. Collectively, this work demonstrates an improved ability to resolve mixed species of Hg in complex samples, establishing HERFD-XAS as an important tool for understanding the environmental and biological cycling of Hg.

## Advancements with HERFD-XAS in metalloprotein studies

As previously discussed, selected main group and transition metals are among the recognized twenty-five essential elements of life. These ‘biometals’ form a critical component of the modern proteome, which itself has been shaped by the geochemistry of the Earth and the changing availability of raw ingredients in the environment. Throughout the evolution of life, organisms have adapted to existing inorganic equilibria and developed sophisticated mechanisms for the acquisition, utilization, and storage of these essential biometals [106]. These systems often contain an assortment of multi-subunit proteins and numerous metal-containing cofactors which perform advanced redox chemistry, converting the Earth’s resources into new chemical forms and storing or producing energy. Inevitably, this presents a challenge to researchers seeking to understand the complex composition and functionality of these systems.

### Manganese in photosystem II

An early example of the capabilities of high resolution XAS techniques for improving our understanding of metals in vital biological molecules is illustrated in the ongoing investigation of the Mn_4_Ca cluster in photosystem II (PS II). Embedded in the thylakoid membrane of oxygenic photosynthetic organisms (i.e. plants, cyanobacteria, and algae), PS II is a multi-subunit pigment protein complex which catalyzes the water oxidation reaction at the active site of the oxygen-evolving complex (OEC) [107,108]. Across the early 2000s, multiple X-ray crystal structures were generated for the PS II complex containing the Mn_4_Ca cluster in cyanobacteria with resolutions between 3.2 Å and 3.8 Å [109–112]. However, structures at this resolution did not permit accurate determination of the positions of Mn and Ca, the bridging and terminal ligands, nor the Mn—Mn/Ca bond distances.

Alongside the limitations of resolution, the Mn_4_Ca cluster has been observed to be highly susceptible to radiation damage, making it essentially impossible to obtain meaningful data with the X-ray dose and temperatures usually employed during XRD data collection [113]. Alternatively, XAS experiments require a lower X-ray dose than XRD and allow greater control in monitoring radiation damage [70,114]. However, obtaining the EXAFS spectra of systems containing multiple metals is challenging as the presence of the rising edge of other elements inhibit the ability to resolve bond distances for the metal of interest [115]. This presents an issue for studies of PS II, as the Mn_4_Ca cluster is accompanied by a non-heme Fe on the electron acceptor side of the plastoquinone oxidoreductase complex [116]. The solid-state detectors commonly utilized for conventional XAS experiments are typically limited to resolutions of around 150-200 eV at the Mn K-edge, making discrimination of the Mn Kα (at 5898 eV) from that of the Fe Kα fluorescence (at 6404 eV) challenging [114].

In 2005, Yano and colleagues demonstrated alleviation of this issue using a high resolution spectrometer design, employing a monochromator system with multiple Ge(333) crystals to probe a subset of 2*p*$ \to $ 1*s* transitions (at ∼5899 eV) of the Mn Kα fluorescence line [114]. This new detection technique was found to (i) improve the metal-metal bond distance resolution by 0.09 Å, and (ii) permit more precise determination of the number of metal-metal moieties. Where earlier EXAFS studies of the S1 and S2 catalytic states for the OEC reported Mn—Mn distances of ∼2.7 Å and ∼3.3 Å [117], and proposed the presence of two or three di-oxo-bridged Mn_2_ motifs [118], the high resolution EXAFS data generated by Yano *et al*. determined Mn—Mn bond distances of 2.73 Å and 2.81 Å in a 2:1 ratio, providing evidence of the presence of three Mn—Mn moieties in the S1 and S2 states of the Mn_4_Ca cluster [114]. Whilst this study employed an early analyzer spectrometer design to record the HERFD-XAS, it demonstrates a key advantage of the technique in its ability to separate fluorescence lines, ultimately improving spectroscopic resolution [84]. With the refinement of the experimental design, and the aid of complementary methods, HERFD-XAS has the potential to provide novel insights into the local coordination and speciation of metals in biomolecules.

### Nitrogenase: the FeMoco cofactor

#### The molybdenum and iron oxidation states

Returning to the previous nitrogenase example, further contributions to understanding the Mo—Fe metallocluster have been accomplished with aid of advanced X-ray spectroscopic methods, particularly through the partnership of valence-to-core X-ray emission spectroscopy (VtC XES) and HERFD-XAS. In 2002, crystallographic analysis of the Mo-base nitrogenase lead to the identification of a central light atom within the FeMoco cluster. This was postulated to be a light atom such as C or N, though definitive assignment could not be made within the resolution of the crystal structure [119]. Further investigations combining EXAFS and nuclear resonant vibrational spectroscopy (NRVS) corroborated the existence of this central atom but failed to determine its identity [120,121]. In 2011, Lancaster and colleagues collected Fe VtC XES data on (i) the complete MoFe protein containing FeMoco and an eight-Fe P-cluster, (ii) isolated FeMoco in N-methyl formamide (NMF), and (iii) a gene-deletion mutant of the MoFe protein containing only the P-cluster [122]. The experimental data showed a significant intensity at 7101 eV only present with relation to the FeMoco active site. Correlation of protein data to model complexes was used to empirically assign the Kβ’’ feature to a carbide, with DFT calculations on FeMoco corroborating this assignment as a central C atom [122]. Published simultaneously with the XES study, X-ray diffraction data obtained by Spatzel *et al*. at the unprecedented resolution of 1.0 Å clearly demonstrated that the observed electron density from the central light atom was most consistent with C. This study also utilized pulsed magnetic resonance and enrichments with the stable magnetic isotopes ^13^C and ^15^ N which definitively showed the presence of a central carbide in the intact enzyme [123]. However, informed mechanistic discussions cannot proceed without knowledge of electronic structure. Thus, efforts have since turned to understanding the oxidation state distributions of Mo and Fe in FeMoco.

Recent progress in our understanding of the electronic structure of Mo within the FeMoco cluster has been achieved with HERFD-XAS, offering reprieve from the large core-hole lifetime broadening seen in conventional Mo K-edge spectra. A 2013 study conducted by Lima *et al*. showed that TD-DFT could be used with substantial accuracy to predict energies and intensities of Mo K-pre-edge features and demonstrated attainment of higher resolution Mo K-edge spectra with HERFD-XAS for a range of Mo model compounds in variable ligand environments [124]. With adequate HERFD-XAS model spectra and an established TD-DFT protocol, Lima and co-workers utilized these combined experimental and theoretical approaches in 2014 to postulate the oxidation state of the Mo cofactor of FeMoco in Mo-dependent nitrogenase as best described as Mo(III) [125]. This is an intriguing finding which contrasts the universally accepted assignment of Mo(IV), with Mo(III) previously unreported in biology, which may warrant reconsideration of the assignment of Fe oxidation states in the cofactor. However, some additional interesting discrepancies also remain. Both the Mo and V nitrogenases have an S = 3/2 ground state in the resting enzyme, and the XAS of the V enzyme is very clearly consistent with a V(III) oxidation state [126], with V(III) being isoelectronic with Mo(IV). Thus, either the formal oxidation states of the Fe atoms differ between the V and Mo enzymes, or the assignment of Mo(III) might be misleading. Moreover, the ^95^Mo hyperfine coupling of the Mo enzyme observed by magnetic resonance is very small [127,128], which would be more consistent with an even formal oxidation state.

Elucidation of the Fe oxidation states has proven intrinsically challenging with conventional X-ray spectroscopic methods given the multitude of Fe atoms existing in the protein; 8 within the P-cluster [8Fe:7S] and 7 within FeMoco [Mo:7Fe:9S: C] [59]. The combination of several Fe XANES studies by Lee *et al*. (1998), [129], Corbett *et al*. (2006) [130], and Fay *et al*. (2011) [131], generated qualitative discussions of oxidation state differences between the P-cluster and FeMoco but yielded no definitive assignment of Fe oxidation states. Elucidation has since been achieved by Wenke *et al*. in 2019, utilizing spatially resolved anomalous dispersion (SpReAD) refinement, a combination of X-ray crystallography and XAS which produces site-specific X-ray absorption spectra. Assuming [MoFe_7_S_9_C]^−^ overall charge, this study found the FeMoco oxidation state to be the average of three Fe(II) (ferrous Fe1, Fe3 and Fe7) and four Fe(III) (ferric Fe2, Fe4, Fe5 and Fe6) [132], representing a localization of Fe oxidation states which contrasts the delocalization observed in large Fe—S clusters [133,134]. Although this does not provide definitive confirmation of the oxidation environment, this accomplishment illustrates the strengths of combinatorial X-ray techniques when effectively designed, whilst also serving as a reminder to the limitations of conventional XAS when used in isolation.

#### Selenium as a selective probe

In 2017, a partially localized electronic structure for the FeMoco unit was calculated from broken symmetry quantum mechanics/molecular mechanics (QM/MM) studies by Benediktsson and Bjornsson [135]. This provides some support to the highly localized electronic structure proposed from the SpReAD study, but raises questions about the true degree of localization/delocalization in the structure. Though the SpReAD technique provided innovative insight, it utilized single crystals and thus offered a select set of states for observation. This limitation and the generated uncertainty surrounding electronic structure has guided motivation for the development of selective probes which can interrogate the local electronic state of FeMoco, extending beyond the resting state to reduced forms and catalytic intermediates. Henthorn *et al*. explored this idea in a 2019 study which utilized selective substitution of S by Se in FeMoco, affording a unique probe for the interrogation of local Fe—Se interactions with HERFD-XAS. This provides a notable advantage over previous approaches and techniques as introducing a non-native element to the nitrogenase FeMoco cofactor creates a selective probe for X-ray spectroscopy, addressing the issue of convolution with other moieties in the protein structure including the P-cluster, F-cluster and other requisite components which collectively contain many S atoms, prohibiting precise elucidation with S XAS [136].

**Figure 9.**
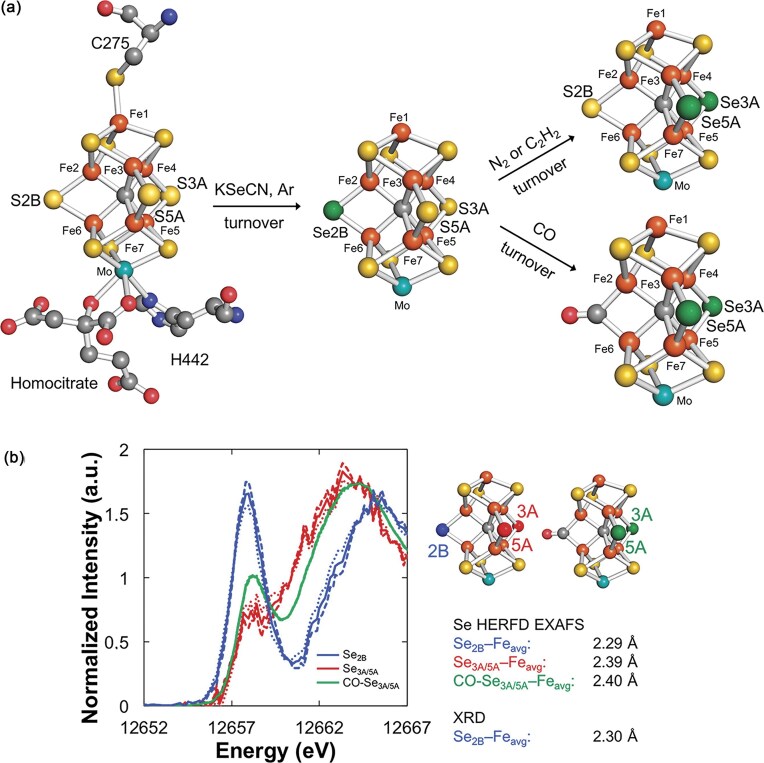
Results from investigations using Se as a selective probe and substitution for S in Femoco. **(a)** Schematic of Se incorporation and migration reactions occurring within the Femoco cluster derived from A. vinelandii (PDB-IDs 3U7Q, 5BVG, 5BVH). Unlabelled atoms are depicted as follows; N (blue), O (red), and C (grey). **(b)** Deconvoluted experimental Se K-edge HERFD-XAS spectra (left) of Se_2B_ (blue), Se_3A/5A_ (red), and CO-Se_3A/5A_ (green). The dashed lines are deconvolutions using Av1Se_lo_/Av1Se_reac_, the dotted lines using Av1Se_hi_/Av1Se_reac_, and the solid lines the average of the two. Corresponding average Se−Fe distances extracted from Se HERFD EXAFS data are also presented (right). AvSe_lo_, Se-incorporated Femoco prepared from 250 μM KSeCN solution; AvSe_hi_, Se-incorporated Femoco prepared from 10 mM KSeCN solution; AvSe_reac_, Se-incorporated Femoco prepared from 10 mM KSeCN solution and inhibited with CO via reaction for 20 min. [Reproduced under the Creative Commons Attribution 4.0 International license from ref. 136 © 2019 American Chemical Society].

According to Spatzel and colleagues, the addition of KSeCN to the Mo-dependent nitrogenase of *A. vinelandii*, under proton-reducing turnover conditions (argon atmosphere), results in incorporation of Se at the bridging positions of the FeMoco cluster. Substitution was found to initially occur at the 2B position, although migration to the 3A and 5A positions takes place under turnover in the presence of a non-proton substrate (N_2_ or acetylene), or via exposure to CO, creating a CO bridge between Fe2 and Fe6 (Fig. 9a) [137]. Although this substitution offers selectivity and precise targeting—with the choice of positions for the substituted Se in FeMoco allowing for selective interrogation of bridging interactions—the achievable energy resolution in conventional Se XAS is notably lower than that of S XAS, due to the significantly shorter 1*s* core-hole lifetime and subsequent broadening in Se K-edge spectra [100]. As we have discussed, this limitation can be overcome using HERFD-XAS with high resolution Bragg optics and selective measurement of the Kα_1_ fluorescence line (2*p*_3/2_$ \to $ 1*s*) to suppress spectroscopic broadening.

Results from the HERFD-XAS studies of Se-substituted nitrogenase by Henthorn *et al*. indicate that the 2B and 3A/5A bridging positions of FeMoco are electronically distinct and that these positions are perturbed upon CO binding as a cofactor (Fig. 9b) [136]. In these investigations, the electron paramagnetic resonance (EPR) spectra of Se-substituted FeMoco revealed a broadened S = 3/2 signal, qualitatively alike to native FeMoco, verifying that minimal perturbation of the electron structure occurs with Se substitution. The Fe2/Fe6 edge demonstrated evidence of an antiferromagnetically coupled diferric pair, supported by (i) TD-DFT calculations on a fictitious [Fe_2_SeS]^n+^ (n = 0, +1, +2) series for which analogous S and Se K-edge XAS spectra were calculated within a single complex, (ii) comparisons of HERFD-XAS spectra for a reference dimer complex [Et_4_N]_2_[Fe_2_Se_2_(SPh)_4_], and (iii) an observed 1.5 eV shift and greater pre-edge intensity in the Se Kα_1_-edge spectra for the 3A/5A nitrogenase [136].

The assignment of oxidation state and relative coupling for Fe2/Fe6 are consistent with the SpReAD study [132] and QM/MM calculations [135], respectively. Alternatively, the Fe3/Fe4/Fe5/Fe7 face of the cofactor was found to exhibit a more localized ferrous character, suggesting an average Fe oxidation state of +2.25 to +2.5 in the resting state [136]. This result could corroborate either study, however, limited conclusions may be drawn given the similar occupancies of the 3A and 5A positions observed in this study, though future investigations may be able to achieve determination providing successful preparation of samples with sufficiently disparate 3A and 5A populations.

The functionality of Se as a selective probe for the investigation of enzymatically relevant metalloclusters is anticipated to expand alongside the development of advanced spectroscopic methods. This is demonstrated by Henthorn and DeBeer, in their recent exploration of Se Kβ VtC XES and Se Kβ HERFD-XAS in reduced Se-containing compounds and Fe—Se dimers. The Se Kβ fluorescence line corresponds to the 3*p*$ \to $ 1*s* decay and subsequent 4*p*$ \to $ 1*s* emission events. Due to spin-orbit coupling, the mainline transitions—which are sensitive to relative Se oxidation state—may be divided into Kβ_1_ (3*p*_3/2_$ \to $ 1*s*) and Kβ_3_ (3*p*_1/2_$ \to $ 1*s*) transitions. Additionally, the Kβ_2_ (4*p*$ \to $ 1*s*) represents the valence-to-core transition, dominated by bonding interactions of the Se photoabsorber [138]. Se VtC XES showed sensitivity to distinct covalent bonding interactions, evidenced by differences in the spectra of Li_2_Se, [Et_4_N][SeH], and KSeCN. However, Se VtC XES spectra of [Fe_2_Se_2_]^m+^ dimers demonstrated only minor sensitivity to changes in the Fe oxidation state, though Se Kβ HERFD-XAS was found to exhibit comparatively greater sensitivity. Subsequent computational studies indicate that both Se VtC XES and Kβ HERFD-XAS are sensitive to Se protonation in Fe—Se complexes, leading to the postulation that a combined approach may provide a powerful tool for the elucidation of protonation/alkylation in Se-substituted Fe—S clusters [138].

### Cubane iron-sulfur clusters

Most recently, Grunwald and colleagues have utilized a combination of conventional Fe K-edge XAS and S K-edge HERFD-XAS to interrogate the evolution of the Fe—S bond covalency during oxidation state changes in cubane Fe—S clusters or [4Fe—4S] [139]. As one of the most ubiquitous metallocofactors, Fe—S clusters are the driving force behind many essential biological processes including Fe homeostasis, multielectron transfer and enzymatic catalysis, as highlighted in the previous examples of FeMoco and P-clusters in nitrogenases [140–143]. Specifically, cubane or cuboidal [4Fe—4S] clusters are abundant and versatile, acting as mediators of long-range electron transfer or furnishing the active sites of enzymes in many biological systems. In natural enzymatic systems, [4Fe—4S] clusters undergo reversible redox changes, accessing [4Fe—4S]^0/1+/2+/3+^ oxidation states, to accomplish electron transfer with low energy barriers and high efficiency [142,143]. This chemical functionality affords valence isomerism and a high density of states within the [4Fe—4S] structure, making these systems challenging to study [139].

Elucidation of the redox states and the inorganic core structures in [4Fe—4S] systems have been enabled using well-defined synthetic [Fe_4_S_4_(SR)_4_]*^n−^* molecular analogues containing thiolate ligands (SR), however, Grunwald *et al*. were the first to establish a full synthetic series of all oxidation states accessible by 1-electron transformation of the Fe atoms utilizing the same ligand set [143]. In 2022, the group reported a complete biomimetic Fe—S cubane redox series, using the K*_n_*[Fe_4_S_4_(DmpS)_4_] model structure where *n* = 0-4 and 2,6-dimesitylphenyl (Dmp) acts as a stabilising ligand. Characterization and oxidation state assignment was initially accomplished using a combination of EPR, XRD, UV-visible electronic absorption spectroscopy, cyclic voltammetry, and ^57^Fe Mössbauer spectroscopy [143], the latter of which is a powerful technique which probes individual Fe atoms but requires challenging isotopic enrichment [139,144,145]. In a subsequent 2024 study, XAS was recognized as an appropriate alternative to provide qualitative oxidation state assignments in bulk samples [139]. Ligand K-edge XAS has been demonstrated in previous studies as a valuable tool to probe the covalency of a metal-ligand bond, as intensity of the ligand pre-edge feature is proportional to the mixing of ligand orbitals and metal *d* orbitals [146,147].

Both the Fe K-edge XAS and S K-edge HERFD-XAS spectra were collected at the European Synchrotron Radiation Facility (ESRF) in Grenoble, France. Fe K-edge XAS spectra were collected at the BM23 beamline utilizing a Si(111) double-crystal monochromator with flat Si mirrors positioned at 3.0 mrad for harmonic rejection, recording spectra in transmission mode [139]. S K-edge HERFD-XAS spectra were collected on the ID26 beamline using a cryogenically cooled Si(111) double-crystal monochromator with mirrors used to achieve a spot size of 200 × 100 μm². The first harmonic of the undulator source was used, with higher harmonics rejected using Si-coated mirrors. HERFD-XAS measurements were collected at the maximum of the Kα emission line and utilized a crystal array spectrometer composed of six cylindrically bent lithium niobate (LiNbO_3_) crystals with a 1 m radius of curvature [139]. All samples were presented as finely ground powders at room temperature under an inert atmosphere (argon) protected within air-tight sample bags or between S-free Kapton foil [139].

**Figure 10.**
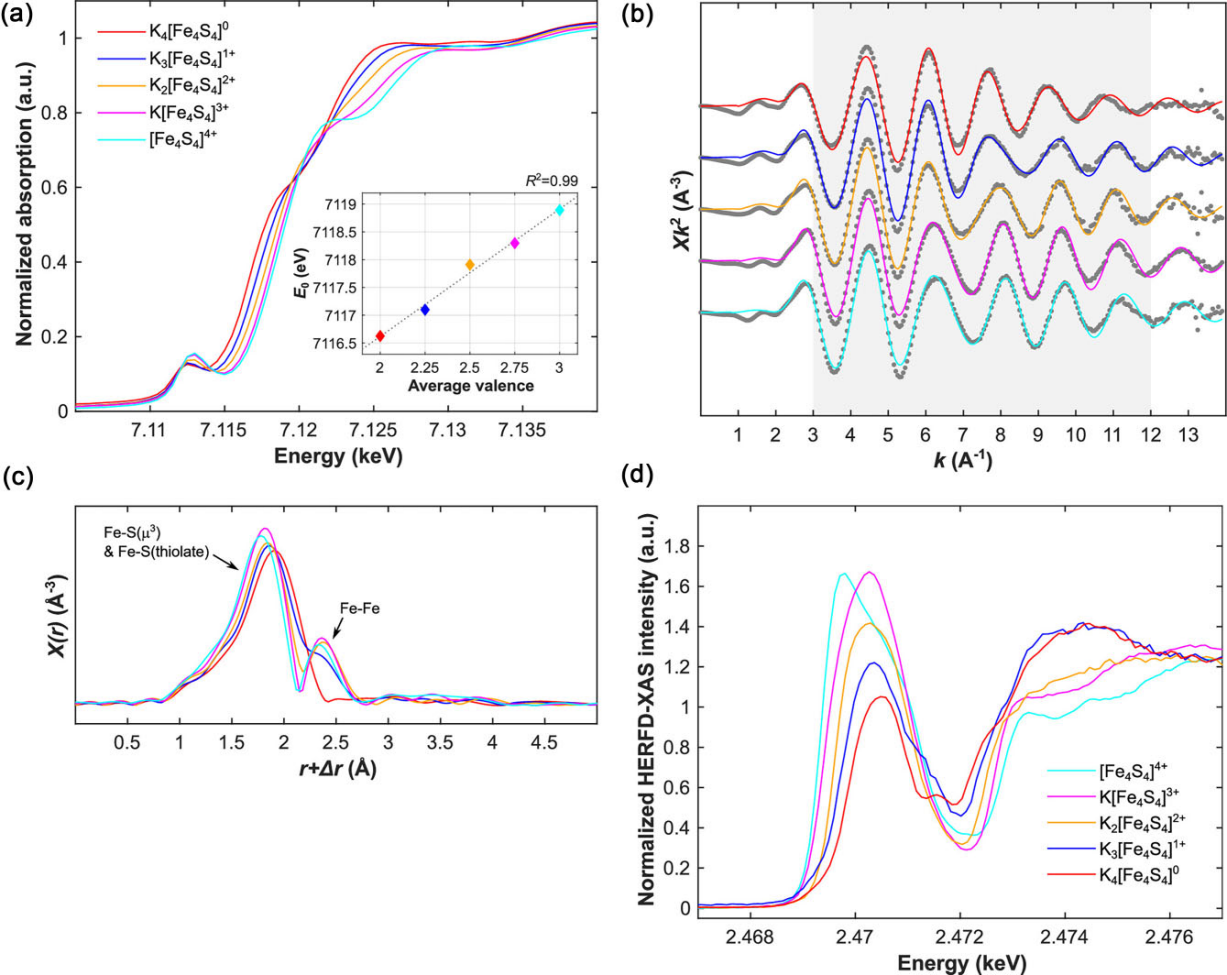
Fe K-edge XAS and S K-edge HERFD-XAS data for Kn[Fe_4_S_4_(DmpS)_4_] (*n* = 0-4) powdered samples measured at room temperature. **(a)** Normalized Fe K-edge XAS spectra. The inset shows the correlation between the average Fe oxidation state (average valence) and the energy of the inflection point (${E_0}$); a linear fit of these data is presented as a dotted gray line, indicating an increase in ${E_0}$ of 0.57 ± 0.03 eV per 1-electron oxidation. **(b)** Unfiltered EXAFS spectra (gray dots) in *k*-space, and the corresponding fits (colored lines). The range over which the fit was carried out is shaded in gray. **(c)** FT-EXAFS spectra (*k*^2^-weighted). **(d)** Normalized S K-edge HERFD-XAS spectra. [Reproduced under the Creative Commons Attribution 4.0 International license from Ref. 139 © 2024 American Chemical Society].

The Fe K-edge XAS spectra of the K*_n_*[Fe_4_S_4_(DmpS)_4_] (*n* = 0-4) model series demonstrated a typical structure of pseudo-tetrahedrally coordinated high-spin Fe atoms, with pre-edge 1*s*$ \to $ 3*d* transition at ∼7112 eV and shoulder at ∼7120 eV. These features, and the mid-edge transition, diminish in intensity and shift to lower energy as the [4Fe—4S]*^n^*^+^ oxidation state reduces from *n* = 4 to *n* = 0 (Fig. 10a) [139]. Previous structural analysis of the nitrogenase Fe protein revealed that this decreased pre-edge is associated with reduced electric-dipole 3*d*-4*p* mixing which is influenced by oxidation state and the ligand field symmetry [148]. In the calibration series of Fe K-edge XAS spectra, the shift of the absorption edge energy (${E_0}$) with changing oxidation state, was determined to be (i) 0.57 ± 0.03 eV or (ii) 0.59 ± 0.08 eV, depending on the applied method: (i) the position of the inflection point of the absorption edge, or (ii) the shift of the midpoint of the smoothed step-function. The oxidation assignment was also reflected in the Fourier-transformed EXAFS spectra of the cubane series (Fig. 10b and c), with identified decreasing amplitude and broadening of the first- and second-shell peaks as [4Fe—4S]*^n^*^+^ was further reduced [139].

Even at room temperature, S K-edge HERFD-XAS provided notably greater energy resolution of the pre-edge structure compared to conventional XAS for a range of standards measured in previous studies [149–151], permitting meaningful interpretation of these attributes in relation to metal-ligand covalency for the K*_n_*[Fe_4_S_4_(DmpS)_4_] (*n* = 0-4) model series [139]. Both of the S K-edge HERFD-XAS pre-edge peak main lines, including the formally forbidden S 1*s*$ \to $ψ* (sum of antibonding orbitals of [4Fe—4S]) transitions of the sulfide and thiolate S atoms (∼2470 eV) [149,150], and the pure dipole-allowed S 1*s*$ \to $ S 3*p* transition (∼2474 eV), visibly shift to higher energies upon reduction (Fig. 10d) [139]. This aligns with existing literature which suggests that reduction of the complex results in a lower effective nuclear charge for Fe, increasing the binding energies of *d*-electrons and translating to higher energies for these pre-edge transitions [148,151]. These results corroborate with ^57^Fe Mössbauer studies which suggested that all redox events were predominantly Fe-centred [143]. However, the covalencies calculated from this data (using the intensity of the 1*s*$ \to $ψ* pre-edge transition) indicate that total covalency increases from [4Fe—4S]^0^ to [4Fe—4S]^3+^, but plateaus upon superoxidation to [4Fe—4S]^4+^. Notably, this discontinuity was not revealed in ^57^Fe Mössbauer, Fe K-edge XAS or UV-visible electronic absorption spectroscopy but may be associated with the break in the trend of Fe—Fe bond distances identified by single crystal XRD [139]. While there this no current evidence to demonstrate the involvement of the all-ferric [4Fe—4S]^4+^ cluster in biological processes [143,152–154], Grunwald *et al*. suggest that these new findings may hint towards a unique role for this cluster which may extend beyond electron/proton transfer [139].

This study represents a significant feat and a noteworthy development in our understanding of cubane Fe—S clusters, made possible using ligand K-edge HERFD-XAS. Furthermore, the establishment of methods to characterize the Fe K-edge energy across a complete [4Fe—4S]*^n^*^+^ redox series, further highlights the utility of XAS as an alternative or complementary technique to other spectroscopic methods and may inspire routine application of this approach in future metalloprotein studies [139].

### Zinc metalloproteins as spike protein targets

In another recent example of the application of HERFD-XAS to metalloprotein studies, Dolgova and co-workers utilized Zn Kα_1_ HERFD-XAS to investigate the binding of the severe acute respiratory syndrome coronavirus 2 (SARS-CoV-2) spike protein to the human enzyme angiotensin-converting enzyme 2 (ACE2) [155]. Formally a carboxypeptidase containing Zn^2+^ at the active site of the protease domain, ACE2 is the initial target of the SARS-CoV-2 spike protein [156,157]. Prior studies interrogating the protein using fluorescent probes have suggested that ACE2 exists as a monomer in the plasma membrane and that binding of the SARS-CoV-2 trimeric spike protein to the receptor binding domain (RBD) does not induce oligomerization of ACE2 [158]. However, data obtained from other studies utilizing mass photometry demonstrate that this 1:1 model is overly simplistic and an inadequate representation of the *in vivo* situation [155,159].

Many previous studies have ignored the presence of Zn^2+^ in the ACE2 active site and/or neglected to evaluate its potential role in this protein-protein interaction. In some reported SARS-CoV-2 spike receptor-binding structures, Zn^2+^ has been completely omitted. In other cases, the coordination around Zn^2+^ has been poorly postulated, with some studies reporting no coordinated atoms at distances closer than 3.8 Å [160], three-coordinate sites missing activated water ligands [161,162], and structures with unrealistic bond-angles [161]. Notwithstanding these inconsistencies, utilizing the enhanced spectroscopic resolution of Zn Kα_1_ HERFD-XAS, Dolgova and co-workers identified that binding of the spike RBD to dimeric ACE2 causes a distortion of the protein active site relative to the other forms (i.e. monomeric ACE2 with/without spike RBD, and dimeric ACE2 without spike RBD) [155].

Zn Kα_1_ HERFD-XAS spectra were collected at SSRL on beamline 15-2 using a Si(311) double-crystal monochromator, with harmonic rejection achieved at the cut-off energy of ∼18 keV using upstream Rh-coated optics, and a seven-element array of spherically bent Si(642) crystal analyzers (θ_B_ = 81.4°) in Johann geometry on a 1 m Rowland circle [155]. Conventional Zn K-edge XAS spectra were collected on beamline 7-3 using a Si(220) double-crystal monochromator with harmonic rejection accomplished by detuning the monochromator to 60% of the peak intensity. X-ray absorption was measured simultaneously with the Zn Kα_1_ fluorescence excitation spectrum, using an array of 30 discrete Ge detectors [155]. For both beamlines, the incident X-ray energy was calibrated to the first inflection point of a Zn metal foil (9660.7 eV). Samples were maintained at 10 K and inclined at 45° to the incident X-ray beam to give a path length of 2.8 mm [155].

**Figure 11.**
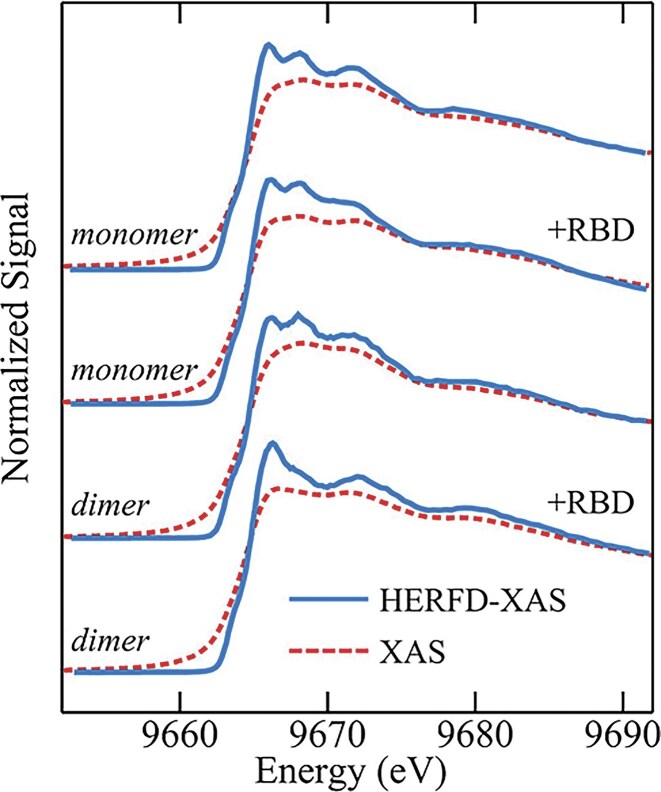
Near-edge conventional Zn K-edge XAS spectra (red broken lines) and Zn Kα_1_ HERFD-XAS spectra (solid blue lines) for monomeric and dimeric ACE2 with (+RBD) and without spike RBD. [Reproduced with permission from ref. 155; Dolgova, NV, Qureshi, M, Latimer, M, *et a*l. Structural changes at the zinc active site of ACE2 on binding the SARS-CoV-2 spike protein receptor binding domain, *Inorg Chem*, 2025;**64**: 3831-41. © 2025 American Chemical Society].

A change in coordination environment is evident from the distinct near-edge structure of dimeric ACE2 with RBD in the Zn Kα_1_ HERFD-XAS spectrum, compared to the other spectra (Fig. 11). This is supported by EXAFS analysis and DFT calculations which suggest that binding of the RBD to dimeric ACE2 induces a subtle conformational change which is likely accompanied by a change in the coordination of a glutamate residue from a monodentate to a bidentate ligand [155]. Notably, this alteration in coordination was not discernible in the near-edge spectra using conventional Zn K-edge XAS, where all ACE2 spectra appeared essentially identical (Fig. 11). This new data highlights the interesting level of flexibility possessed by the dimeric form of ACE2, which may offer a more robust model for the study of ACE2-spike interactions than the previous monomeric ACE2 model [155].

## Utility of HERFD-XAS in mammalian biology and human health

As previously discussed, many metal ions are essential to life, and hence, critical to the maintenance of good health, with deficiencies resulting in potential growth disorders, severe biological malfunctions, carcinogenesis, or death [36,163]. Conversely, an excess of these metal ions may result in toxicity as the redox activities of many coordination metals (i.e. Fe, Cu, Mn) can become unrestrained when metal homeostasis is disrupted. This dyshomeostasis or inhibition of regulatory mechanisms (i.e. metal transport and sequestration) can lead to the inappropriate binding of metals to protein sites and subsequent toxicity and oxidative stress [164]. Furthermore, organisms are susceptible to toxicity resulting from exposure to non-essential heavy metals such Hg and lead (Pb) [36]. Here we will discuss examples where HERFD-XAS has been utilized to explore the molecular fates of essential and non-essential metals and metalloids in mammalian samples with improved sensitivity at highly dilute concentrations.

### Understanding the exposure routes of mercury

#### Accumulation of mercury in hair

Resuming the discussion of the role of HERFD-XAS in providing improved speciation analysis for Hg, here we present recent research investigating the link between the chemical form of Hg and the route of exposure in humans. Given the natural biogeochemical cycling of Hg, some degree of human exposure is inevitable. Most commonly we experience methylmercury exposure via the consumption of high trophic-level fish [165], however, the general population may also be exposed to medical treatments which contain ethylmercury as a preservative [166], Hg-containing dental amalgams and related products of degradation [167–169], and inhalation of Hg vapor from damaged household appliances such as fluorescent lamps [170]. The concentration of Hg in blood or urine is often used to monitor Hg intake, although scalp hair is the commonly chosen matrix for methylmercury analysis. Typical concentrations of Hg in hair samples are 0.1 to 3.0 ng/mg [171], significantly below the minimum concentration of ∼1 ppm Hg required for mass-independent and mass-dependent fractionation isotopic analysis [172]. These concentrations are also below the feasible limits for speciation analysis with conventional X-ray techniques and present ambiguity in identifying coordinating ligands [173].

Recent investigations by Manceau *et al*. have demonstrated detection of Hg in hair at concentrations as low as 0.5 ppm, with identification of specific coordination to C, N, and S ligands, using Hg Lα_1_ HERFD-XAS [172]. In this work, Manceau and co-workers examined hair samples with known sources of Hg, incorporated metabolically (*in vivo*) or exogenously (*in vitro*), in one of four forms; methylmercury (MeHg), ethylmercury (EtHg), elemental mercury (Hg^0^) and aqueous mercury (Hg^2+^). Hair samples containing MeHg as an *in vivo* source were obtained from (i) residents of Pará in Brazil, exposed via consumption of fish from local contaminated rivers, and (ii) residents of Grenoble, France with no known exposure apart from average fish consumption. Hair containing Hg^0^ as an *in vivo* source was obtained from residents of France with dental amalgams collected within the year following the filling procedure. Hair samples contaminated with EtHg and Hg^2+^ were prepared *in vitro* via treatment with thimerosal and via simulation of Hg vapor over 20-days, respectively [172].

Hg Lα_1_ HERFD-XAS spectra were measured in high energy resolution fluorescence yield detection mode at the ESRF using a Si(111) double-crystal monochromator with horizontal and vertical palladium (Pd)-coated mirrors. The Hg Lα_1_ fluorescence line was selected using five spherically bent Si(555) analyzer crystals aligned at θ_B_ = 81.8° in a vertical Rowland geometry, with diffracted intensity measured by a Si drift diode detector in single photon counting mode, providing a 3*d*_5/2_ core-hole width of ∼3.0 eV [172].

**Figure 12.**
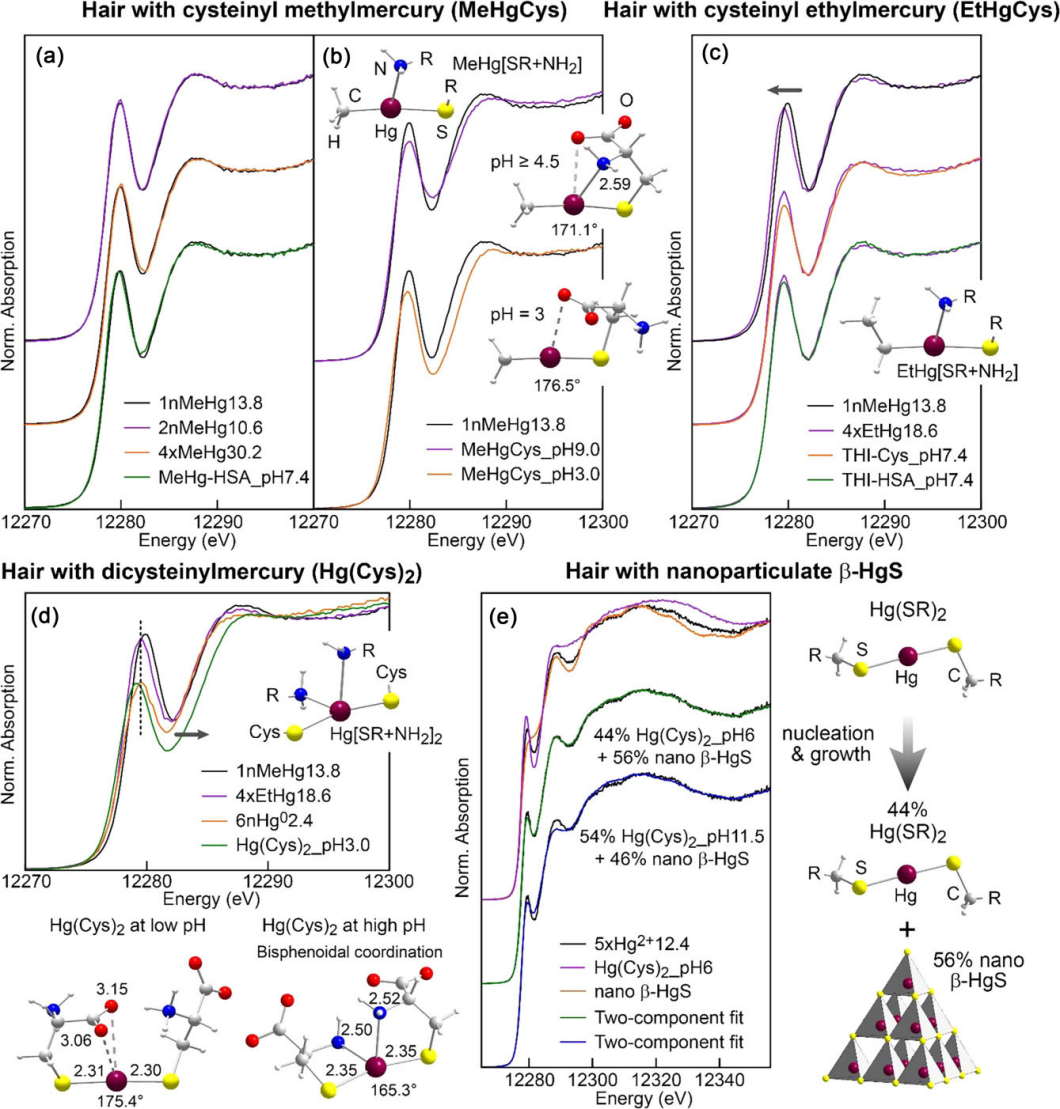
Hg coordination in hair derived from HR-XANES spectra. **(a)** and **(b)** Methylmercury (MeHg) in hair (1nMeHg13.8; 2nMeHg10.6; 4xMeHg30.2) and MeHg complexed with human serum albumin (MeHg-HSA) and L-cysteine at pH ≥ 4.5 (MeHgCys) as reference. **(c)** Ethylmercury (EtHg) in hair (4xEtHg18.6) and thimerosal complexed with L-cysteine or HSA (THI-Cys and THI-HSA). **(d)** Hair containing endogenous inorganic Hg from dental amalgams (6nHg02.4) compared to Hg complexed with L-cysteine at and above physiological pH. The dashed vertical line is a guide for the position of the near-edge peak. Structures for Hg(Cys)_2_ at low and high pH are depicted (below). At acidic pH, the protonated amine group (NH^3+^) of L-cysteine is nonbonding, indicated by a shift (arrow) to higher energy of the HR-XANES trace (green) beyond the intense near-edge peak. **(e)** Hair containing exogenous inorganic Hg (5xHg2 + 12.4) against reference spectra of Hg(Cys)_2_ and nanoparticulate metacinnabar (β-HgS). The schematic (right) represents a reaction in which linear Hg(SR)_2_ units are transformed to β-HgS. Bond lengths (Å); bond angles (black); Hg (dark red); S (yellow); N (blue); O (red); C (grey); H (light grey). [Reproduced with permission from Ref. 172; Manceau, A, Enescu, M, Simionovici, A, *et al*. Chemical forms of mercury in human hair reveal sources of exposure, *Environ Sci Technol*, 2016;**50**: 10721-29. © 2020 American Chemical Society].

The obtained Hg Lα_1_ HERFD-XAS spectra indicated equivalent MeHg bonding when assimilated into hair *in vivo*, as seen in samples 1nMeHg13.8 and 2nMeHg10.6 from Brazilian residents, or *in vitro* when complexed with human serum albumin (MeHg—HSA) or L-cysteine (MeHgCys). In each case, the HERFD-XAS spectra showed evidence of MeHg bonded to a thiol (at 2.35 Å), methyl (at 2.07 Å), and a nearby N-containing ligand in the hair (at 2.6 Å), which is protonated (—NH_3_^+^) and non-bonding at low pH but available at pH $ \ge \,\,$4.5 according to optimized molecular structures (Fig. 12a and b) [172]. Spectra showed that EtHg was also bonded to a thiol S and a N atom in hair (4xEtHg18.6), alike to thimerosal complexed with L-cysteine or HSA (THI-Cys and THI-HSA) (Fig. 12c). EtHg in hair was characterized by a $ - $0.5 eV shift on the near-edge peak relative to MeHg, as verified by XANES calculations for computed structures of EtHgCys and MeHgCys at high pH. The coordination of organomercury compounds in hair were denoted MeHg[SR+N] and EtHg[SR—N] [172]. The spectra generated from hair sourced from patients receiving dental amalgams (6nHg^0^2.4) showed features indicative of coordination to two S and one or two N atoms (Fig. 12d), giving a Hg[(SR)_2_N_1-2_] structure. Hair samples exposed to aqueous Hg^2+^ (5xHg^2+^12.4) were found to have statistically identical spectra (Fig. 12e), though distinct from other samples, with linear combination fits identifying two major components: 44$\,\, \pm \,\,$6% Hg(Cys)_2_ at pH 6 and 56$\,\, \pm \,\,$6% nanosized β-HgS [172].

These results show that it is possible to distinguish between multiple species of Hg in hair using HERFD-XAS and an appropriate reference database and demonstrate distinction between Hg speciation and the route of exposure. This approach could be applied to the identification of other known or putative toxic metals in human tissues and presents potential applications in forensics when coupled with nanoimaging techniques to track the timing of exposure events [174].

#### Organometallic mercury in the brain

In 2022, James *et al*. conducted further studies on Hg speciation in human tissue, using HERFD-XAS to investigate the chemical forms of Hg and Se in brain tissue. Their results reveal a dramatic difference in the molecular fate of Hg between individuals who suffered acute organometallic Hg exposure (poisoning) and individuals who experienced chronic low-level exposure from a diet rich in marine fish [89]. Experiments were carried out at the SSRL, with a similar arrangement to previously described HERFD-XAS configurations implemented by Nehzati and co-workers [74,90], utilizing a Si(311) double-crystal monochromator and a seven element array of spherically bent Si crystal analyzers, with Si(555) and Si(844) utilized for Hg Lα_1_ and Se Kα_1_ emissions, respectively. Samples were cooled to 10 K using a He flow cryostat and mounted at 45^o^ to the incident X-ray beam [89].

**Figure 13.**
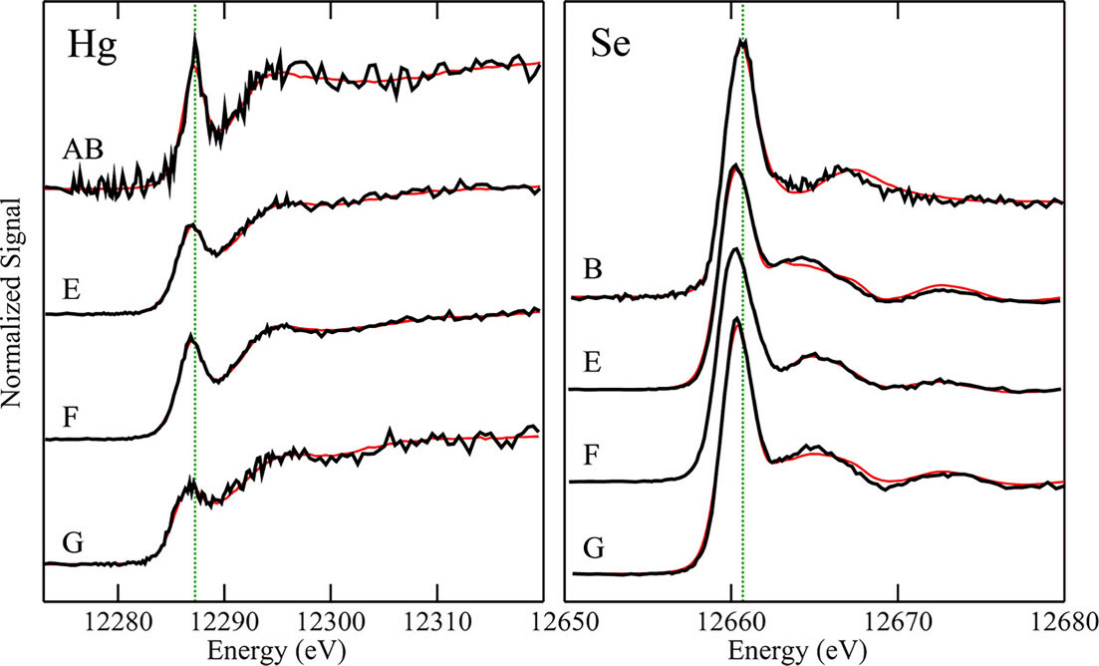
Hg Lα_1_ (left) and Se Kα_1_ (right) HERFD-XAS of brain tissue samples. Sample AB is the weighted average of the two data sets A and B. The black lines show experimental data, and the red lines show the linear combination analyses of Hg species (HgSe, RS—Hg—SR, RS—Hg—CH_3_) and Se species (HgSe, R—Se—R’, RS—Se—SR, R—Se—SR, oxidised Se) as briefly described in text. Green broken lines are included to emphasize differences in peak positions. Data was acquired on the SSRL SPEAR3 storage ring on beamlines 6-2. [Reproduced with permission from Ref. 89; James, AK, Dolgova, NV, Nehzati, S, *et al*. Molecular fates of organometallic mercury in human brain, *ACS Chem Neurosci*, 2022;**13**: 1756-68. © 2022 American Chemical Society].

Linear combination fitting of Hg Lα_1_ HERFD-XAS data from samples A and B, representing chronic low-level exposure via fish consumption, revealed almost exclusive presence of Hg in its organometallic form, particularly MeHg coordinated to S (likely a cysteine thiolate). These samples were derived from lifetime residents of the Republic of Seychelles (East Africa) with diets comprising a sizeable proportion of marine fish. It has been reported that Seychellois have brain Hg concentrations up to 10-fold higher than populations with lower dietary Hg, although with substantial variation between individuals [175,176]. Samples E, F, and G were derived from individuals who experienced instances of organometallic Hg poisoning. These cases of short-term high-level exposure resulted in both short-term survival (E and F) and long-term survival (G). In contrast to samples A and B, these tissues were found to contain a more complex mixture of Hg species including thiolate bound MeHg (RS—Hg—Me), mercuric selenide (HgSe), and inorganic Hg bound to two thiolate donors (Hg(SR)_2_) [89]. Hg Lα_1_ HERFD-XAS spectra of these samples are shown in Fig. 13.

**Figure 14.**
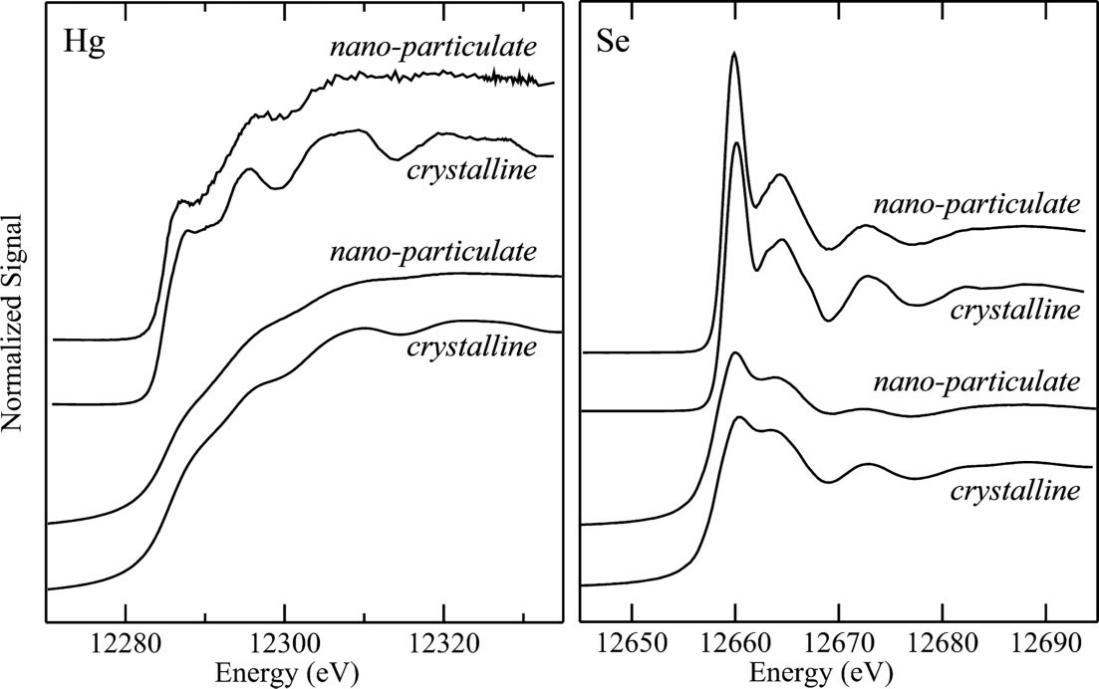
Comparison of HERFD-XAS (top pairs) and conventional XAS (bottom pairs) of nanoparticulate and crystalline HgSe for Hg (left) and Se (right). Data was acquired on the SSRL SPEAR3 storage ring on beamlines 7-3 (XAS) and 6-2 (HERFD-XAS). [Reproduced with permission from Ref. 89; James, AK, Dolgova, NV, Nehzati, S, *et al*. Molecular fates of organometallic mercury in human brain, *ACS Chem Neurosci*, 2022;**13**: 1756-68. © 2022 American Chemical Society].

Previous conventional XAS studies have demonstrated subtle spectral differences between crystalline and nanoparticulate mercuric selenide (nano-HgSe) [177,178], however, Hg Lα_1_ and Se Kα_1_ HERFD-XAS spectra obtained by James and co-workers illustrate notable enhancement of spectroscopic resolution for both elements and both forms of HgSe (Fig. 14), though there is disparity in the amplitudes of the post-edge structure, particularly for the crystalline HgSe. The Hg Lα_1_ HERFD-XAS spectra for nano-HgSe also shows reasonable agreement with previous data collected by Manceau and co-workers [179]. However, linear combination analysis of Hg Lα_1_ and Se Kα_1_ spectra for samples E, F and G shows some discrepancy in the characterization of the form of HgSe, depending on the element selected for analysis. Despite the enhanced spectroscopic resolution, the specific thiolate donors within the Hg(SR)_2_ coordination environment could not be determined from Hg Lα_1_ HERFD-XAS spectra.

Furthermore, efforts to examine the possible presence of methylmercury L-selenocysteinate in samples containing appreciable levels of thiolate-bound MeHg (A, B, E and F) were unsuccessful, with minimal improvement in fits and poor correspondence between Hg Lα_1_ and Se Kα_1_ HERFD-XAS [89]. As previously stated, these results illustrate noteworthy connections between the molecular fate of Hg and the route and severity of exposure, however, at the same time they emphasize the lingering challenge of determining structure and connectivity beyond the central unit—a challenge to which HERFD-XAS is not a faultless solution.

### Selenium in bovine cartilage

Revisiting Se Kα_1_ HERFD-XAS, there have been recent ground-breaking improvements in the detection limits of this essential element in biological matrices. As previously emphasized, elucidating the speciation of ultra-dilute elements with conventional XAS has presented an immense challenge, obstructed primarily by poor signal-to-noise ratios in XAS spectra. In 2019, Bissardon and colleagues reported the first direct analysis of the speciation and concentration of Se in bovine articular cartilage [180]. The ability to identify and quantify the speciation of dilute elements presents an enormous benefit to metallomics research, and in this case, provides an opportunity to advance our understanding of Se biotransformation and incorporation into cartilage matrix [180]. These investigations are relevant to human health in the context of the growing body of evidence which suggests that the pathogenesis of osteoarthritis may be associated with Se deficiency and subsequent oxidative stress conditions [181–183]. In this study, cartilage explants from immature bovine metacarpophalangeal joints cultured for 21 days were utilized as *in vitro* models [184]. These explants were snap-frozen in hexane and cryo-milled into a homogenous fine powder which was transferred to a He cryostat (∼10 K) under anoxic conditions for HERFD-XAS analysis.

Se Kα_1_ HERFD-XAS spectra were collected at the 6 GeV ESRF storage ring on the CRG-FAME beamline (BM30b) operating with a Si(220) double-crystal monochromator and an array of five spherically bent Ge(844) crystal analyzers in Johann geometry, equipped with a downstream Si drift detector [180]. The total Se concentrations for samples were determined by triple quadrupole inductively coupled plasma mass spectrometry (ICP-QQQ), reporting concentrations in the range of 100-200 ppb for dried immature and mature cartilage samples, and below 60 ppb for cartilage exposed to hydrated conditions. These samples did not yield HERFD-XAS spectra of satisfactory quality for interpretation. However, HERFD-XAS measurements were obtained for cartilage cultured in standard serum-free medium supplemented with insulin-transferrin-selenium (ITS) containing 6.7 μg/L sodium selenite, in the presence or absence of growth factors (FT), or further supplemented with 50 μg/L sodium selenite [180].

**Figure 15.**
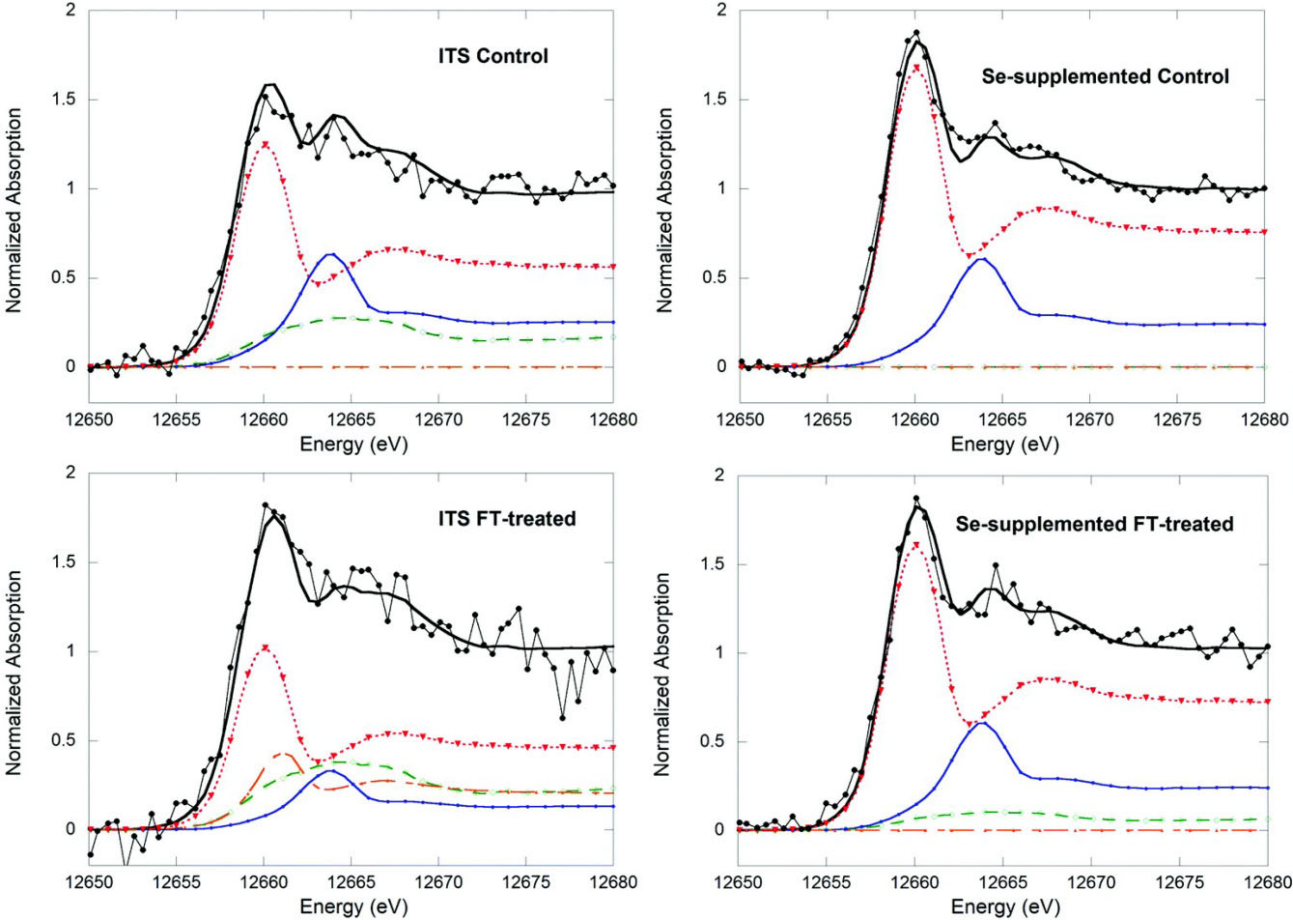
Se Kα_1_ HERFD spectra of model Se compounds for cultured cartilage explants matured *in vitro* for 3 weeks in media supplemented with ITS (left), with both ITS and sodium selenite for (Se-supplemented, right), in absence (control, top) or presence of growth factor (FT-treated, bottom). Data was acquired on the ESRF storage ring on the CRG-FAME (samples and solid references) and CRG-FAME-UHD (liquid references) beamlines. Up to 90 replicate scans were necessary for dilute cartilage samples to achieve a sufficient signal-to-noise ratio. Traces indicate the cartilage sample data (black connected data points), the fit sum (black solid line), and the components sodium selenite (blue line), GPX (green line), selenocysteine (orange line), and selenodiglutathione (red line). [Reproduced with permission from Ref. 180; Bissardon, C, Proux, O, Bureau, S, *et al*. Sub-ppm level high energy resolution fluorescence detected X-ray absorption spectroscopy of selenium in articular cartilage, *Analyst*, 2019;**144**: 3488-93. © 2019 The Royal Society of Chemistry].

Interpretable HERFD-XAS spectra were produced from total Se concentrations as low as 400-500 ppb in ITS control and FT-treated cartilage. Linear combination fitting of experimental spectra suggested a thioselenide species (i.e. selenodiglutatione, R—S—Se—S—R) as the major component in both Se-supplemented samples (∼70%) and ITS samples (45-55%), with sodium selenite forming a minor component (12-23%) in all cases (Fig. 15). The metabolic interpretations of these results are discussed by Bissardon *et al*. [180], though the most noteworthy outcome of this investigation is the advancement towards improved speciation of highly dilute elements using HERFD-XAS. These findings emphasize the contrast in the achievable signal-to-noise ratio, and subsequent detection of dilute species, between conventional XAS and HERFD-XAS methods.

However, this study also highlights an inconsistency in the experimental design of HERFD-XAS, with Bissardon *et al*. employing an alternative crystal analyzer configuration to Nehzati and co-workers. Crucially, as previously stated in the discussion of crystal analyzer systems, the use of Ge(844) crystals for Se Kα_1_ HERFD-XAS does not satisfy the near-90° Bragg condition required for optimum energy resolution. Thus, while the advantages of the technique for the detection of ultra-dilute Se were evident in this study, the spectra collected by Bissardon *et al*. demonstrate only marginal improvement in spectroscopic resolution relative to conventional Se K-edge XAS. In contrast, the use of a Si(844) crystal analyzer system by Nehzati and co-workers demonstrated a near 2-fold resolution improvement compared to conventional XAS [90], which better represents the full capabilities of HERFD-XAS for both enhanced spectroscopic resolution and background rejection, collectively.

This combined improvement in energy resolution and signal-to-noise has also been demonstrated in a 2025 study of Se in biological tissues which investigated the metabolic fates of selenite and selenotrisulfides in rodent kidneys, liver, testes, and blood [185]. Like the prior work by Nehzati (2021) and James (2022), Se Kα_1_ HERFD-XAS spectra was collected on the SSRL 15-2 beamline with an array of seven spherically bent Si(844) crystal analyzers. Notably, the Se Kα_1_ HERFD-XAS model spectrum obtained for the non-native selenotrisulfide treatment compound (penicillamine selenotrisulfide) showed a marked enhancement in spectroscopic resolution compared to the spectrum collected using conventional Se K-edge XAS [185]. Furthermore, the application of Se Kα_1_ HERFD-XAS permitted the analysis of Se speciation in a variety of dilute samples (e.g. 0.28-0.44 ppm Se in blood plasma) and revealed distinct changes in the composition of selenospecies between red blood cells and plasma, highlighting the blood as the critical site for metabolic transformations of inorganic Se to selenotrisulfides and subsequent bioactivity prior to distribution into other tissues [185].

## HERFD-XAS in drug design and pharmacological research

Where our previous discussions have focused on HERFD-XAS applications for human health from a toxicological perspective, we now examine the use of HERFD-XAS in drug design and subsequent pharmacological studies, aiding to improve our understanding of the complex biochemistry of drug compounds for the treatment of human disease. There are currently limited examples of HERFD-XAS studies for this purpose, however, the with the growing recognition of this technique, it is anticipated that HERFD-XAS will make valuable contributions in future investigations of metal-related therapeutic agents and metal-based drugs. Subsequent discussions will focus on the former application, examining advancements in our understanding of the action of therapeutic agents devised to counter disease-related metal ion dyshomeostasis through sequestration.

### Understanding metal chelators

#### Copper(II)-binding 8-hydroxyquinolines

Existing since the 1870s [186], 8-hydroxyquinoline (8HQ) and its derivatives comprise a family of lipophilic metal ion chelators which have been historically utilized for a range of applications, most commonly as gravimetric and colorimetric reagents [186–188], and as antiseptics, disinfectants, pesticides, and antifungal/antiprotozoal drugs [189–191]. More recently, CQ (clioquinol; 5-chloro-7-iodo-8-hydroxyquinoline) and PBT2 (5,7-dichloro-2-[(dimethylamino)methyl]-8-hydroxyquinoline) have been postulated as candidate drugs for the treatment of neurological diseases which are hypothesized to be associated with metal dyshomeostasis [192–194]. However, the affinity of 8HQs for binding to divalent metals in solution, and the resulting mechanism(s) of action, remain poorly understood [195].

**Figure 16.**
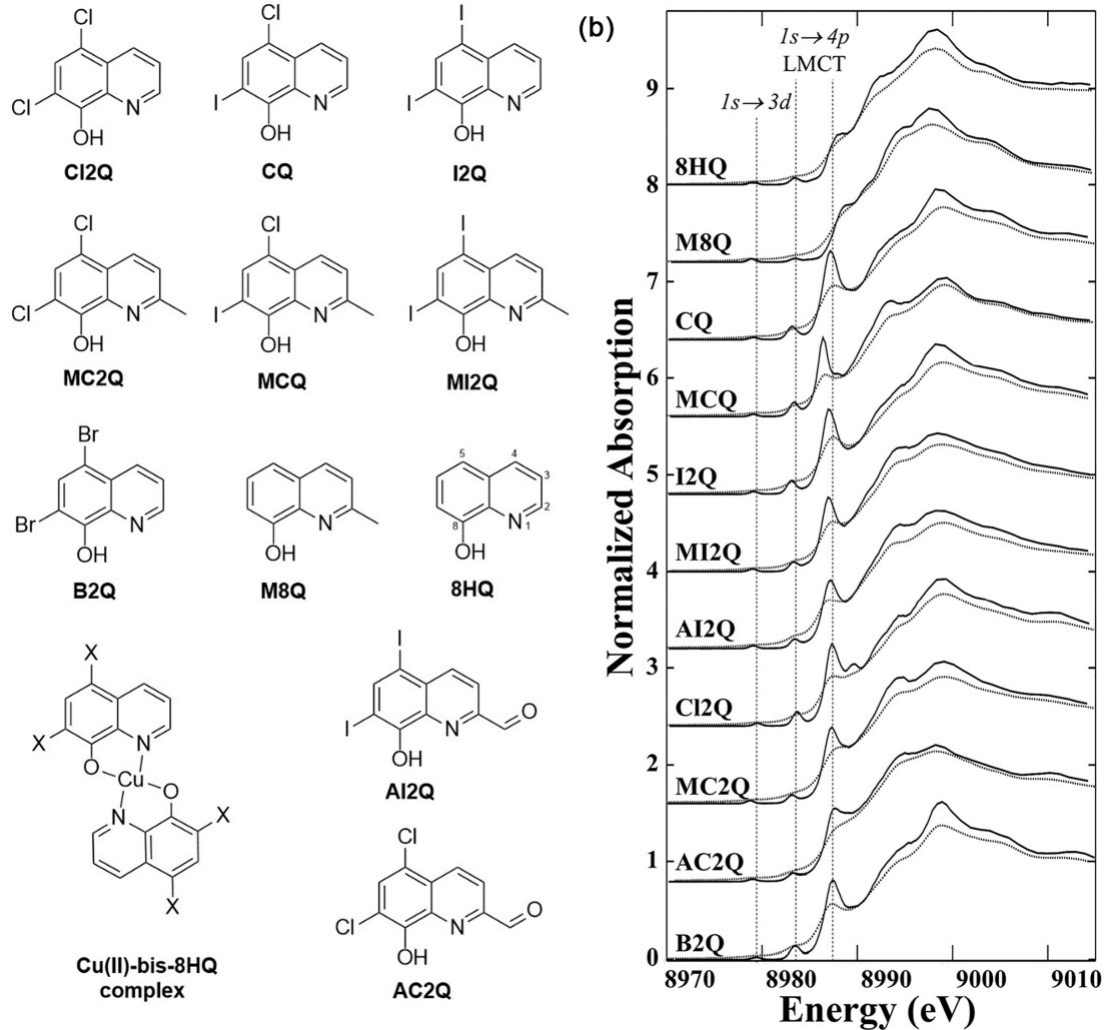
Cu K-edge XAS of Cu(II) complexes with 8HQ and its derivatives. **(a)** Structures of examined 8HQ derivatives and depiction of the square planar-like geometry predicted upon binding of two bidentate 8HQs to Cu(II) (bottom left). **(b)** Experimental Cu(II) near-edge spectra from XAS (broken line) and HERFD-XAS (solid line) of 8HQ and studied derivatives. The broken vertical lines indicate near-edge transitions. A spline was fit to the near-edge spectra and normalized to 1. Spectra are offset vertically by 0.8 for clarity. Data was acquired on the SSRL SPEAR3 storage ring on beamlines 7-3 (XAS) and 6-2 (HERFD-XAS). [Reproduced with permission from Ref. 195; Summers, KL, Pushie, MJ, Sopasis, GJ, *et al*. Solution chemistry of copper(II) binding to substituted 8-hydroxyquinolines, *Inorg Chem*, 2020;**59**: 13858-74. © 2020 American Chemical Society].

A comprehensive investigation of the solution chemistry of a range of Cu(II) binding 8HQs (Fig. 16a) was conducted by Summers *et al*. in 2020, employing a combination of XAS, HERFD-XAS, EPR and UV-visible absorption spectroscopies [195]. As a preface to our discussion of this work it should be noted that previous investigations of the structure of Cu(II) bound CQ (Cu(II)-CQ) using X-ray crystallography in 2004 suggest the coordination of two CQ to Cu(II) via phenolate and N donors, creating a square planar-type geometry [196]. However, XAS data produced by Chen *et al*. in 2007 instead proposed an octahedral coordination environment involving six O or N ligands [197]. These conclusions are contested by the XAS analysis conducted by Summers and colleagues, providing evidence of an approximately square-planar Cu(II) species in solution with CQ [198]. These contradictory results emphasize the difficulty of studying these complexes which exhibit low solubility and redox sensitivity, with Cu(II) prone to photoreduction in the X-ray beam unless appropriate precautions are taken [41,195].

Summers and colleagues collected HERFD-XAS measurements at SSRL on the SPEAR3 storage ring, selecting a subset of 2*p*_3/2_$ \to $ 1*s* transitions of the Cu Kα_1_ fluorescence line. The experimental setup employed a Si(311) double-crystal monochromator with ∼0.3 eV energy resolution, upstream aluminum filters and a Rh-coated mirror for harmonic rejection (∼18 keV cut-off energy), and an array of six Si(444) crystal analyzers in Johann geometry [195]. Stock solutions of a range of 8HQs were prepared by dissolving powders in room temperature surfactant solutions containing 400 mM dodecyl trimethylammonium bromide (DTAB) at pH 7.4. Aqueous Cu(II) 8HQ samples were then prepared from these stocks (diluted to 4 mM) and CuCl_2_ (at a final concentration of 2 mM) which were sonicated, flash frozen in isopentane, and cooled with LN_2_ in acrylic sample cuvettes. During HERFD-XAS measurements, samples were cooled to 10 K using a He flow cryostat and mounted at 45^o^ to the incident X-ray beam [195]. Conventional Cu K-edge spectra were also collected at the SSRL at 10 K on the SPEAR storage ring with a Si(220) double-crystal monochromator [199].

The Cu Kα_1_ HERFD-XAS spectra of these samples were compared to conventional Cu K-edge XAS spectra, highlighting subtle yet valuable differences. As anticipated, all spectra in Fig. 16b show a small 1*s*$ \to $ 3*d* transition at ∼8979 eV which is indicative of Cu(II), however a similarly small peak at ∼8983 eV is well-resolved only in HERFD-XAS spectra [195]. It is possible that this ∼8983 eV feature may arise from the photoreduction of Cu in the beam, yielding Cu(I) with a K-edge at ∼8978 eV, however, HERFD-XAS scan rates are rapid (1-2 minutes) and no photoreduction was observed in conventional XAS scans without scan-to-scan sample translation. Alternatively, the 8983 eV peak and its more prominent neighboring peak at 8987 eV may be tentatively assigned as 1*s*$ \to $ 4*p*_z_ and 1*s*$ \to $ 4*p*_xy_ + ligand-to-metal charge transfer (LMCT) shake-down transitions [195]. These transitions are classified as one-photon two-electron events which occur when metal orbitals shift to lower energies as a result of the effective increase in nuclear charge from the 1*s* photoexcitation in the K-edge [195,200]. The observation of LMCT shake-down transitions is generally thought to be indicative of tetragonal or square-planar type coordination geometry for Cu(II) as the ligand field splitting of the 4*p* manifold results in lower energy peak positions [201,202]. In the series of 8HQ Cu(II) complexes, it was found that 1*s*$ \to $ 4*p* + LMCT shake-down transitions were generally prominent with the presence of this near-edge feature aligning with complexes which were hypothesized to be planar [195]. This agrees with the spectroscopic interpretations of a pseudo square-planar coordination for Cu(II)-*bis*-CQ in previous conventional XAS studies [198].

Interpretations of near-edge features exclusively observed in high energy resolution Cu Kα_1_ spectra, combined with EXAFS fitting and analysis, suggested that the 5,7-dihalogenated 8HQ conformers can also bind Cu(II) in a square-planar-like geometry, with two bidentate 8HQ ligands (Fig. 16a) [195]. EPR analysis and DFT-based calculations support the assignment of Cu(II)-*bis*-complexes with 8HQ, B2Q, CQ, I2Q, and Cl2Q as highly planar, with minor deviations in coordination geometry generating unstable higher-energy structures [195]. EXAFS data suggests that Cu(II)-8HQ complexes exhibit co-planarity and high symmetry. Additionally, it was determined that substituents in the 2-position of 8HQs are capable of modifying the Cu(II) coordination geometry as evidenced by the reduction in intensity of 1*s*$ \to $ 4*p* + LMCT shake-down transitions in the near-edge of 8HQ Cu(II) complexes methylated at the 2-position, and the damped EXAFS amplitudes at high $k$ range indicating reduction of constructive interference from distant halogen backscatters and non-linear Cu(II)—O—X paths [195].

These results highlight the ability to tune the properties of novel metal-bound drug complexes through the careful selection of substituents, allowing for potential improved drug efficacy either via modifying (i) halogen substituents at 5- and 7-positions to tune hydrophobicity or (ii) substituents at the 2-position to tune planarity. This is particularly crucial for potential drug compounds (like 8HQs) which are largely hydrophobic yet must cross the blood-brain barrier to provide the desired therapeutic effect [195].

#### The coordination geometry of PBT2

PBT2[199], the aforementioned analogue of CQ, reportedly exhibits distinctive behavior in clinical trials [203–205], which suggests a more complex mechanism of action beyond the simple Cu(II) sequestration of other Cu(II)-8HQ complexes. With combined data from conventional Cu K-edge XAS, Cu Kα_1_ HERFD-XAS, and EPR methods, Summers *et al*. proposed a novel Cu(II)-*bis*-PBT2 complex with asymmetric coordination which appears to co-exist with the symmetric four-coordinate pseudo square-planar Cu(II)-*bis*-PBT2 complex [199]. This assertation primarily stems from the HERFD-XAS spectra of Cu(II) with PBT2 which revealed a splitting of the 1*s*$ \to $ 3*d* transition into two peaks at ∼8979 eV and ∼8980 eV, suggesting the presence of two different coordination environments. Additionally, the EXAFS curve fitting and results, and findings from previous studies [206,207], suggest that PBT2 may initially bind Cu(II) as a tridentate ligand as opposed to its generally anticipated bidentate behavior, with varying mixtures of Cu(II) PBT2 complexes forming in solution depending on the Cu(II)/PBT2 ratio [199]. These conclusions led researchers to speculate a potential link between the observed effectiveness of PBT2 in clinical trials, particularly its increased ionophoric activity, and its ability to act as a tridentate chelator for Cu(II) and permeate the blood-brain barrier more efficiently than other 8HQs [199].

### New avenues for pharmacological applications

Despite the known controversy surrounding the ‘therapeutic chelation’ approach to neurodegenerative disease treatment, particularly Alzheimer’s disease [208,209], and the questionable efficacy of 8HQs as therapeutic agents, the results described above serve as a useful case study regarding Cu chemistry and the interactions of this essential metal with chelators. The venture to understand the coordination of metal-binding 8HQ drug compounds highlights the ability of HERFD-XAS to reveal elusive spectroscopic details which provide valuable information for the determination of transition metal geometry in solution. In the above example, although inferences had previously been made with conventional XAS, the improved resolution of HERFD-XAS was found to greatly assist in removing some of the inevitable ambiguity in the interpretation of spectra. Subsequently, it is anticipated that HERFD-XAS will offer valuable insights into future studies of metal ion coordination and speciation in medicine and disease treatment.

## Summary and outlook

In this article, we have showcased a broad selection of existing and emerging applications of HERFD-XAS in the development of our understanding of metals and metalloids in biology and medicine. In many cases, our discussions intentionally focused on direct comparisons of conventional XAS and HERFD-XAS spectra, to highlight the substantial improvements observed in spectroscopic resolution and signal-to-noise ratio, providing greater potential for elucidating the speciation of metals in complex and dilute biological samples. In doing so, this overview has encompassed HERFD-XAS for metalloprotein studies, the metabolism of essential and toxic metals in mammalian systems, and the design and characterization of metal chelating therapeutics. These are critical areas of research which impact and expand our understanding of human health and disease treatment. Collectively, we hope that our discussions illustrate the value of high resolution XAS methods in metallomics research and encourage the continued development and utilization of HERFD-XAS by both current and future researchers in the field.

Whilst we have highlighted several advantages of HERFD-XAS relative to conventional XAS, we recognize that the development of this technique is ongoing and researchers seeking to use this method will be presented with inevitable challenges. Where the configurations of conventional XAS experiments are well established, HERFD-XAS experiments require more intricate spectrometer designs, employing crystal analyzers in variable configurations which must be optimized and tailored to generate high-quality spectra for each element of interest. Additionally, we must consider strategies to mitigate the effects of radiation damage on biological samples, a persistent hinderance for all X-ray methods. Given the limited expertise regarding HERFD-XAS design, there currently exists a lack of accessibility, with few synchrotron facilities possessing HERFD-XAS capabilities. Subsequently, commissioning these instruments requires a substantial investment of time and resources.

Despite these limitations and owing to the noteworthy accomplishments that we have witnessed so far, we anticipate an expansion in the applications of HERFD-XAS. We have shown that, alike XAS, HERFD-XAS represents a valuable tool in combination with XRD and other spectroscopic techniques such as VtC-XES and EPR, particularly for the elucidation of metal coordination and binding to proteins and ligand molecules. Most notably, we have illustrated the significant improvements in the energy resolution of HERFD-XAS spectra, relative to conventional XAS, for multiple elements of interest (particularly Hg and Se), demonstrating how the technique can permit discrimination between previously indistinguishable absorption edges, whilst offering considerable background rejection for samples containing low analyte concentrations. This opens new avenues for the study of elements in biological samples at ultra-dilute concentrations, which may be applied to the investigation of the physiological effects of metal species in plants [210], and in understanding the environmental health effects of heavy metals as part of multimodal detection strategies [52], collectively yielding novel insights to metallomics research across all kingdoms of life.

## Data Availability

No new data were generated or analysed in support of this research.
